# Modulation of left ventricular hypertrophy in spontaneously hypertensive rats by acetylcholinesterase and ACE inhibitors: physiological, biochemical, and proteomic studies

**DOI:** 10.3389/fcvm.2024.1390547

**Published:** 2024-09-16

**Authors:** Lucie Hejnova, Zdenka Drastichova, Almos Boroš, Jaroslav Hrdlicka, Michal Behuliak, Jan Neckar, Josef Zicha, Jiri Novotny

**Affiliations:** ^1^ Department of Physiology, Faculty of Science, Charles University, Prague, Czechia; ^2^ Institute of Physiology, Czech Academy of Sciences, Prague, Czechia

**Keywords:** acetylcholinesterase, cholinergic signaling, hypertension, SHR and WKY rats, myocardial proteome, pyridostigmine, trandolapril

## Abstract

**Background:**

The consequences at the molecular level and the mechanisms involved in a possible cardioprotective effect of antihypertensive treatment are not yet fully understood. Here, the efficacy of pyridostigmine (PYR) and trandolapril (TRA) as antihypertensive and antihypertrophic agents was investigated and compared in hypertensive SHR and normotensive WKY rats. In parallel, we investigated the effects of these drugs on myocardial β-adrenergic and cholinergic signaling pathways and protein expression profiles.

**Methods:**

Age-matched male SHR and WKY rats were chronically (8 weeks) treated with PYR or TRA in drinking water. Blood pressure (BP) and heart rate (HR) were monitored telemetrically prior to tissue sampling for biochemical analysis. Baroreceptor reflex sensitivity (BRS) and methylatropine HR response as a measure of vagal tone were evaluated in separate groups of animals.

**Results:**

PYR slightly lowered BP and HR in SHR rats during the dark phase of the day, while TRA effectively reduced BP during the light and dark phases without affecting HR. PYR enhanced BRS and improved vagal tone. There were no significant alterations in myocardial β-adrenergic and cholinergic signaling, with the exception of decreased forskolin-stimulated adenylyl cyclase (AC) activity in SHR rats, which was restored by TRA. Proteomic analysis revealed numerous differences induced by both treatments. Notable were changes in TGFβ-related signaling pathways as well as proteins involved in modifying hemodynamic parameters and cardiac hypertrophy.

**Conclusions:**

PYR is able to slightly decrease BP and HR in SHR rats but effectively increase BRS through vagal potentiation. The specific differences in protein expression profiles in rat myocardium induced by treatment with PYR and TRA reflect different mechanisms of action of these two agents at the molecular level.

## 1 Introduction

Hypertension is one of the main risk factors for ischemic heart disease and heart failure (1). Despite this strong association, there are few experimental studies looking at cardioprotective therapy by potentiating parasympathetic nervous system activity in hypertensive models. The autonomic nervous system (ANS) is a key regulator of blood pressure (BP) and heart rate (HR) in health and disease. ANS imbalance is a common trait in the development of hypertension and cardiovascular disease (CVD), and is characterized by overactivity of the sympathetic nervous system and a deficiency of the parasympathetic nervous system, manifested by an overproduction of epinephrine and norepinephrine with a concomitant deficiency of acetylcholine (2, 3). The progression from hypertension to cardiac hypertrophy is a process that involves remodeling of the myocardium due to increased workload and well as through changes in intracellular signaling and growth regulation that eventually lead to decompensated hypertrophy and heart failure (HF) (4).

The spontaneously hypertensive rat (SHR) is a commonly used model of essential hypertension and cardiac hypertrophy with ANS imbalance. It is an inbred strain developed from the normotensive Wistar-Kyoto rat (WKY) strain (5–7). Originally, SHR and WKY were found to be similar in terms of baroreceptor reflex sensitivity (BRS), i.e., control of HR by the baroreceptor reflex, but with the establishment of hypertension, SHR show a significant reduction in BRS (8). In SHR, the sympathetic arm of BRS (measured as the slope of sodium nitroprusside-induced tachycardia) is preserved, whereas the parasympathetic arm (measured as the slope of phenylephrine-induced bradycardia) is negatively affected (9). Out previous results confirmed a reduced BRS and a shifted ANS balance in the SHR strain (10).

Pharmacological interventions lower blood pressure (BP) by inhibiting the renin-angiotensin-aldosterone system (RAAS), such as angiotensin-converting enzyme (ACE) inhibitors, have long been proven effective in clinical and experimental settings for the treatment of hypertension and HF (11, 12). ACE inhibitors have been shown to be antihypertrophic and cardioprotective in both experimental and clinical settings of HF (13–15). Their cellular mechanisms acting on cardiomyocytes are still largely unexplored. In relation to the ANS, ACE inhibitors act through a central RAAS inhibitory mechanism that reduces sympathetic outflow and decreases the resulting vasoconstriction, thereby lowering BP (16). Furthermore, ACE inhibitors have been shown to reduce cardiac hypertrophy and improve BRS in experimental and clinical settings (17–19).

Due to the central role of the ANS in CVD, pharmacological potentiation of parasympathetic activity appears to be a promising treatment for treatment. The peripherally acting AChE inhibitor pyridostigmine (PYR) has been shown to restore ANS balance by potentiating the parasympathetic nervous system by increasing circulating acetylcholine (ACh) levels, decreasing basal HR and increasing parasympathetic tone (20–22). Notably, PYR has also been reported to enhance BRS in animals with or without HF (20, 23, 24). PYR was found to improve cardiac hemodynamic parameters in Wistar and SHR rats after induction of acute myocardial infarction (AMI), as determined by echocardiography (ECHO) (25–27). Interestingly, treatment with PYR in SHR animals without prior AMI induction was shown to reduce diastolic pressure in addition to mean arterial pressure and ejection fraction (20).

The currently available literature dealing with the cardiac effects of PYR predominantly describes cardioprotective effects of treatments starting shortly after a myocardial infarction (25–31). Only one paper describes chronic treatment prior to myocardial infarction, but the results relate to a post-myocardial infarction time point (26). There is a single paper describing the effects of long-term chronic PYR treatment, but without considaraion of hemodynamics (21). In addition, two papers describe the results of 2-week PYR treatment in WKY and SHR rats either without a focus on hemodynamic parameters or with lower doses (20, 22). Thus, there is a knowledge gap regarding the cardiac effects of chronic PYR treatment in rats without further cardiac interventions (myocardial infarction, heart failure). Furthermore, no one has yet investigated the mechanistic links with the changes in cardiac function induced by chronic PYR treatment.

Although there is a large body of research on the antihypertensive and antihypertrophic effects of trandolapril (TRA) and other angiotensin-converting enzyme inhibitors, the majority of studies have focused on the treatment of heart failure (13–15, 32, 33). One paper on chronic treatment with TRA in SHR focused on myocardial hypertrophy, cardiac fibrosis and ventricular arrhythmias, but the authors did not provide hemodynamic data (34). Therefore, there is similar gap in knowledge regarding TRA as with PYR also exists with regards to TRA. Finally, no one has investigated broader effects of chronic PYR or TRA treatment on the cardiac proteome.

The main aim of our current study was to explore the impact of chronic PYR and TRA treatment on hypertensive (SHR) and normotensive (WKY) rats without further cardiac interventions (induction of myocardial infarction, HF of different etiology). In addition, we also investigated the mechanistic relationships associated with chronic treatments with PYR or TRA. The effects of these treatments on cardiac function, morphology, and proteome were also investigated.

## 2 Materials and methods

### 2.1 Materials

Pyridostigmine and trandolapril were purchased from Sigma-Aldrich (St. Louis, MO, USA) and Mylan (Canonsburg, PA, USA), respectively. [^3^H]QNB was purchased from PerkinElmer, Inc. (Boston, MA, USA), [*α*-^32^P]ATP was from Hartmann Analytic, GmbH (Braunschweig, Germany) and EcoLite liquid scintillation cocktail was from MP Biomedicals (Santa Ana, CA, USA). Aluminum oxide 90 (neutral, activity I) was from Merck (Darmstadt, Germany) and cOmplete protease inhibitor cocktail was from Roche Life Science (Indianapolis, IN, USA). Antibodies against *β*_1_-and *β*_2_-adrenergic receptors were obtained from Santa Cruz Biotechnology (Santa Cruz, CA, USA) and secondary anti-rabbit antibody labeled with horseradish peroxidase were from GE Healthcare (Chalfont St. Giles, UK). Acrylamide and bis-acrylamide from Serva (Heidelberg, Germany) and SuperSignal West Dura chemiluminescent substrate was from Pierce Biotechnology (Rockford, IL, USA). All other chemicals were purchased from Sigma-Aldrich (St. Louis, MO, USA). Nitrocellulose membrane was purchased from Schleicher-Schuell (Erdmannhausen, Germany), Whatman GF/C filters from Whatman Ltd. (Oxford, UK).

### 2.2 Animals and treatment

Groups of approximately 4–5-month-old age-matched WKY and SHR rats were used for the experiments, as SHR rats has developed hypertension and cardiac hypertrophy at this age. The rats were treated for 8 weeks with PYR at a dose of 25 mg/kg/day, or TRA at a dose of 1 mg/kg/day, administered in the drinking water. The animals were housed under standard laboratory conditions (temperature 23 ± 1°C, 12 h light-dark cycle, standard diet, and water *ad libitum*). All experimental procedures were approved by the Ethical Committee of the Institute of Physiology, Czech Academy of Sciences and conformed to the European Convention on Animal Protection and Guidelines on Research Animal Use.

### 2.3 Telemetry measurements

For telemetry measurements of BP and HR, groups of 8 animals were implanted with HD-S10 telemetric transmitters (Data Sciences International, USA) as previously described (10). The animals were allowed a 14-day recovery period, at the end of which basal BP and HR values were recorded for 3 days (5 min intervals were recorded 4 times per hour). Thereafter, BP and HR were monitored continuously for 8 weeks, and final values were obtained during the last 3 days of treatment.

### 2.4 Echocardiography

In separate sets of SHR and WKY rats (*n* = 8–12 animals per group), evaluation of geometrical and functional parameters of the hearts was performed using echocardiographic system GE Vingmed System Seven with 14 MHz linear matrix probe (35). Anaesthesia was induced with 3% of isoflurane (Aerrane, Baxter SA) and then maintained at 2% during the ultrasound procedure. Rectal temperature was maintained within 36.5 and 37.5℃ by a heated table throughout measurements. Diastolic and systolic dimensions of LV were measured during echocardiographic evaluation including anterior and posterior wall thickness (AWTd, PWTd, AWTs, PWTs) and LV cavity diameter (LVDd, LVDs). From these dimensions, the following functional echocardiographic parameters were derived: fractional shortening (FS) = (LVDd −LVDs)/LVDd * 100, ejection fraction (EF) = 100 * (LVDd3 −LVDs3)/LVDd3, diastolic and systolic LV volumes (EDV, ESV) were calculated based on prolate spheroid geometry using the formula EDV = 0.001 * (4 * π/3) * k * LVDd3/8 and ESV = 0.001 * (4 * π/3) * k * LVDs3/8, where (k) is a ratio of long to short axis, stroke volume (SV) = EDV−ESV and cardiac output (CO) = SV * HR, where (HR) is heart rate. Blood flow in the pulmonary artery was assessed by the parameters of peak velocity (Vmax), mean velocity (Vmean), acceleration time (ATp) and ejection time (ETp). Cardiac function was also assessed by mitral flow using the parameters early ventricular filling velocity (E), filling time (FTm), isovolumic contraction time (IVCTm), ejection time (ETm) and isovolumic relaxation time (IVRTm).

### 2.5 Baroreceptor reflex sensitivity and methylatropine HR response

For BRS and methylatropine (MA) response measurements, polyethylene catheters were inserted into the left carotid artery (PE50 for BP measurements) and jugular vein (PE10 for drug infusion) of rats under isoflurane anesthesia (2.5% in air). BP and HR were measured on the second day after surgery using the PowerLab system (ADInstruments, Australia) on partially restrained, conscious animals in transparent measurement boxes (allowing limited movement of the animals). Briefly, the parasympathetic arm of the BRS was determined for each animal as the slope of reflex bradycardia in response to increasing BP elicited by increasing i.v. bolus doses of the α-agonist phenylephrine, as previously described (36). The MA HR response was evaluated in the same animals following administration of 2 mg/kg i.v. of the muscarinic receptor antagonist methylatropine to elicit a rise in HR, as a measure of the involvement of parasympathetic tone in HR control, as previously described (25).

### 2.6 Specimen collection and processing

After treatment, the animals were anesthetized with isoflurane and weighted before killing. Blood was collected from the abdominal aorta with a syringe and centrifuged at 5,000 × g, 4°C for 10 min to obtain plasma. Plasma samples were aliquoted into Eppendorf tubes and frozen in liquid nitrogen. The hearts were excised and weighted. Tissue pieces from the left ventricle and septum were then collected in Eppendorf tubes and frozen in liquid nitrogen. All samples were then stored at −80°C until further analysis.

Samples of the left ventricles of eight animals from each experimental group were prepared for biochemical analysis. Heart tissue sections (approximately 200 mg) were diluted 1:4 with PBS, homogenized twice for 10 s using an Ultra-Turrax homogenizer, and then homogenized for 2 min using a motor-driven glass-Teflon homogenizer (1,200 rpm). For the cholineacetyltransferase (ChaT) assays, 100 µl of the homogenate was centrifuged at 10,000 rpm for 10 min (4°C). The supernatant was aliquoted and frozen in liquid nitrogen. The remaining individual homogenates from each experimental group were pooled into three samples per experimental group. These were as biological replicates. The pooled homogenates were diluted 1:1 with PBS. One part (500 µl) of the homogenate was mixed 1:1 with 100 mM triethylammonium bicarbonate (TEAB) buffer containing 2% (w/v) sodium deoxycholate (SDC), sonicated 3 times for 10 s at 40% efficiency (Bandelin sonicator) and centrifuged at 14,000 rpm for 10 min (4℃). A portion (100 µl) of the supernatant destined for proteomic analysis was diluted with 50 mM TEAB buffer containing 1% SDC to a protein concentration of 1 µg/1 µl and frozen in liquid nitrogen. The remainder of the supernatant was used for SDS-PAGE and Western blotting. The resting homogenates were supplemented with Protease (Complete) and phosphatase (PhosphoSTOP) inhibitors and centrifuged at 800 × g for 10 min (4°C). The supernatant was then centrifuged at 50,000 × g for 30 min (4°C). The resulting pellet was resuspended in TME buffer (20 mM Tris-HCl, 3 mM MgCl_2_, 1 mM EDTA; pH 7.4) and centrifuged again. The final pellet, representing the crude membrane fraction, was dissolved in TME. The diluted crude membranes were aliquoted, frozen in liquid nitrogen, and stored at −80°C. These samples were used for the determination of muscarinic receptors and measurement of AC activity. Protein content in samples was measured by BCA method.

### 2.7 Choline acetyltransferase activity

The individual myocardial samples were diluted with PBS to a protein concentration of 25 µg per 10 µl in the case of SHR and 28 µg per 10 µl in the case of WKY. Ten µl of the diluted homogenates were pipetted into 0.6 microtubes on ice. The reaction was started by adding 10 µl of the incubation buffer (final concentrations: 20 mM sodium phosphate pH 7.4, 200 mM NaCl, 10 mM MgCl_2_, 16 mM EDTA, 0.2 mM serine, 10 mM choline and 0.2 mM AcCoA with [^3^H]AcCoA (60,000 dpm/sample, specific activity 50 dpm.pmol^−1^) and placed on a thermoblock. After 15 min of incubation at 37°C, the reaction was stopped by adding 500 µl of sodium tetraphenylboronate/acetonitrile (5 mg/ml) to the microtube and the microtube was placed in a scintillation vial containing 5 ml of 10 mM sodium phosphate pH 7.4. Then 1.5 ml of sodium tetraphenylboronate/acetonitrile and 10 ml of toluene scintillation solution (0.2 g POPOP and 4 g PPO/1 L toluene) were added to the scintillation vial. The solutions were mixed several times and after phase separation the radioactivity was measured with a scintillation counter (Tri-Carb, Perkin Elmer).

### 2.8 Acetylcholinesterase activity

Plasma acetylcholinesterase activity was determined by a modified Ellmańs assay using the specific AChE inhibitor BW284c51 [BW1,5-bis-(4-allyldimethylammoniumphenyl) pentan-3-one dibromide]. The assay was performed with the Acetylcholinesterase Activity Assay Kit (Sigma-Aldrich, Saint Louis, USA) according to the supplier's instructions.

### 2.9 Adenylyl cyclase activity

Adenylyl cyclase activity was assessed in myocardial crude membrane fractions using the conversion of [α-^32^P]ATP to [^32^P]cAMP. Membrane fractions (20 μg of protein) were incubated in a total volume of 100 μl of reaction mixture (48 mM Tris·HCl, 100 mM NaCl, 2 mM MgCl_2_, 20 mM GTP, 5 mM phosphoenolpyruvate, 40 mM 3-isobutyl-1-methylxanthine, 0.1 mM cAMP and [^3^H]cAMP (∼13 000 counts/min) as a tracer, 3.2 U of pyruvate kinase, and 0.8 mg/ml BSA). Basal and stimulated adenylyl cyclase activity was measured. Stimulation was performed by incubation in the presence of 10 µM isoprenaline, 10 mM NaF or 10 µM forskolin. After 1 min of preincubation, 0.4 mM M ATP was added together with [α-^32^P]ATP (2,000,000 counts/min) and the incubation proceeded for 30 min at 32°C. The reaction was stopped by adding 200 μl 0.5 M HCl and heated to 100°C for 5 min. Samples were neutralized with 200 μl 1.5 M imidazole. Separation of newly formed [^32^P]cAMP was performed by using dry alumina column chromatography. The amount of [^32^P]cAMP and [^3^H]cAMP in eluate were measured using liquid scintillation spectrometry (37). The detected amount of [^32^P]cAMP was corrected for recovery with [^3^H]cAMP, which was 70%–75%.

### 2.10 Determination of muscarinic receptors

Myocardial muscarinic receptors were determined by radioligand binding assay with a muscarinic receptor antagonist [^3^H]-quinuclidinyl benzilate (QNB), as previously described (38). Sixty µg of crude membranes were incubated in 1 ml of incubation buffer (10 mM HEPES, 100 mM NaCl, 10 mM MgCl_2_; pH 7.4) containing increasing concentrations of [^3^H]QNB (0.03–1 nM) for 120 min at 37°C. Nonspecific binding was detected by incubation in the presence of 5 µM atropine. The incubation was terminated by adding 6 ml of ice-cold incubation buffer and rapid filtration through GF/C filters presoaked in 0.3% polyethyleneimine. The filters were additionally washed 2 times with 3 ml of incubation buffer. The radioactivity retained on the filters was counted by liquid scintillation. The results were analyzed using the GraphPad program (GraphPad Software, La Jolla, CA, USA) and B_max_ and K_D_ were determined for each group. The differences between the control group, the pyridostigmine group and the trandolapril group were analyzed using GraphPad Prism 9 (GraphPad software, San Diego, CA, USA).

### 2.11 SDS-PAGE and western blotting

Samples (detergent samples or crude membrane fractions) were solubilized in Laemmli buffer and loaded onto standard 10% or 12% polyacrylamide gels. Electrophoresis was performed at 200 V for 45 min. Resolved proteins were then transferred to a nitrocellulose membrane (Protran BA85, GE Healthcare, Little Chalfont, Buckinghamshire, UK) for 90 min at 100 V, blocked for 30 min with 5% non-fat dry milk in TBS buffer (10 mM Tris and 150 mM NaCl; pH 8.0), and then incubated overnight at 4°C with specific primary antibodies. The next day, the membranes were washed three times for 10 min with TBS buffer containing 0.3% Tween 20 (TBS-T), and the secondary IgG antibody conjugated to horseradish peroxidase (Amersham, Little Chalfont, UK) was applied for 1 h at RT. After washing three times for 10 min with TBS-T, the blots were visualized with the enhanced chemiluminescence technique using the Clarity Western ECL Substrate (Biorad) or the SuperSignal West Femto substrate (Pierce Biotechnology, Rockford, IL, USA). The blots were scanned and quantitatively analyzed using Image Lab Software 6.1 (BioRad). The intensity of the Western blot bands was normalized to total protein content (Ponceau S staining).

### 2.12 Proteomic analysis by nLC-Ms2

The LC/MS analysis of the preparations from left ventricles was performed using nano reversed-phase PepMap C18 chromatography columns (EASY-Spray column, 50 cm × 75 μm ID, 2 μm particles, 100 Å pore size). The buffers of mobile phase A or B were consisted of 0.1% formic acid dissolved in water or acetonitrile, respectively. The loading buffer consisted of 2% acetonitrile, 0.1% trifluoroacetic acid and water and was used to load the samples onto the C18 PepMap100 trap column (5 μm particle size, 300 μm × 5 mm, Thermo Scientific, Waltham, MA, USA). The sample loading was proceeded at 18 μl/min for 4 min. The gradient of mobile phase B from 2% to 35% was used to elute the peptides from the column. The eluted peptide cations were converted to gas-phase ions by electrospray ionization. The analysis was performed by a Thermo Scientific Orbitrap Fusion Mass Spectrometer (Q-OT-qIT, Thermo Scientific), which was set to a resolution of 120 K (at 200 m/z) with an ion count of 1 × 10^6^ to perform survey scans of peptide precursors from 350 to 1,400 m/z. Tandem MS was performed by isolation at 1.5 Th with the quadrupole, higher energy collisional dissociation fragmentation with a normalized collision energy of 35, and rapid scan MS analysis in the ion trap. The maximum injection time was 150 ms, while the MS2 ion count target was set to 10^4^. For MS2, only the precursors with charge states 2–6 were scanned. The dynamic exclusion time was set to 30 s with a tolerance of 10 ppm for the selected precursor and its isotopes. The cycle time was set to 2 s and the selection of the monoisotopic precursor was activated during the measurement.

### 2.13 Data analysis

Raw data analysis and quantification were performed using MaxQuant software (version 1.6.3.4). The false discovery rate (FDR) was set at 1% for peptides and proteins. The minimum peptide length was set to seven amino acids. Searches of MS/MS spectra using the Andromeda search engine were performed in the Rattus norvegicus database containing roughly 29 000 proteins. Quantifications were performed using the label-free algorithm in MaxQuant and data were analyzed in Perseus software. Differences in pairwise comparisons were defined as qualitative or quantitative change. The absence or presence of a protein in an experimental group in pairwise comparisons was defined as a qualitative change from the parallel condition, provided that the protein was detectable or undetectable in at least two of three biological replicates. A difference in protein expression level of at least twofold between two experimental groups was defined as a quantitative change, provided that the protein was simultaneously detected in at least two of three biological replicates. GO enrichment analysis was performed using the DAVID tool (Database for Annotation, Visualization and Integrated Discovery, LHRI, david.ncifcrf.gov). Protein-protein associations were analyzed using the STRING database. Proteins were sorted according to their affiliation with different signaling pathways and figures were generated using BioRender (Biorender.com, Toronto, Canada).

### 2.14 Statistics

Data are expressed as mean ± SEM. Statistical significance was determined by two-way ANOVA followed by the Bonferroni multiple comparison test using GraphPad Prism 9. The differences were considered significant at *p* ≤ 0.05.

## 3 Results

### 3.1 Blood pressure and heart rate

**Figure 1:**
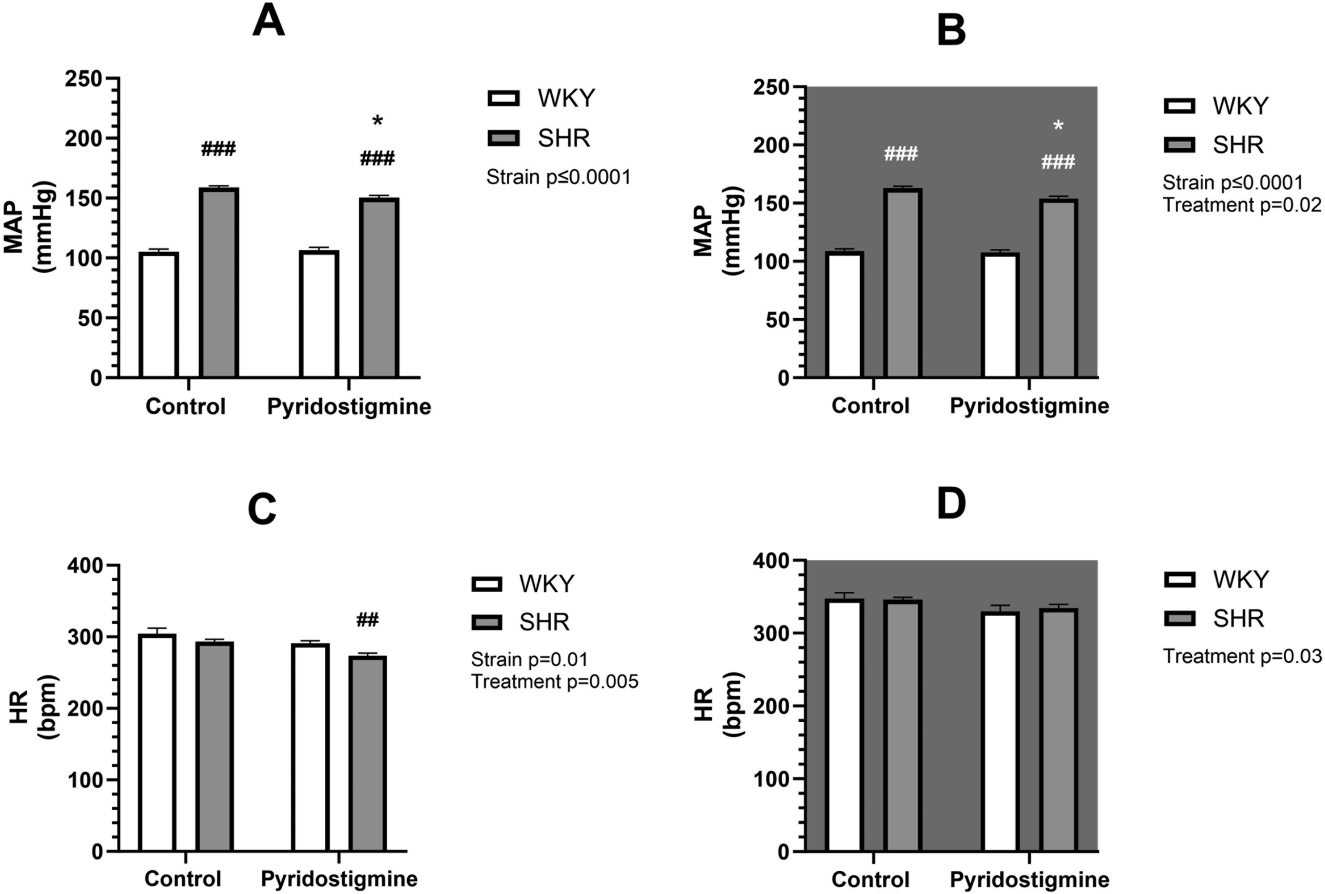
Arterial pressure and heart rate in SHR and WKY rats before and after treatment with PYR. Mean arterial pressure (MAP) **(A,B)** and heart rate (HR) **(C,D)** were recorded telemetrically during the light **(A,C)** and dark **(B,D)** phases. Data are expressed as mean ± SEM (*n* = 8 animals per group). *p ≤ 0.05 vs. corresponding control within the strain; ^##^*p* ≤ 0.01, ^###^*p* ≤ 0.001, vs. untreated WKY group.

**Figure 2:**
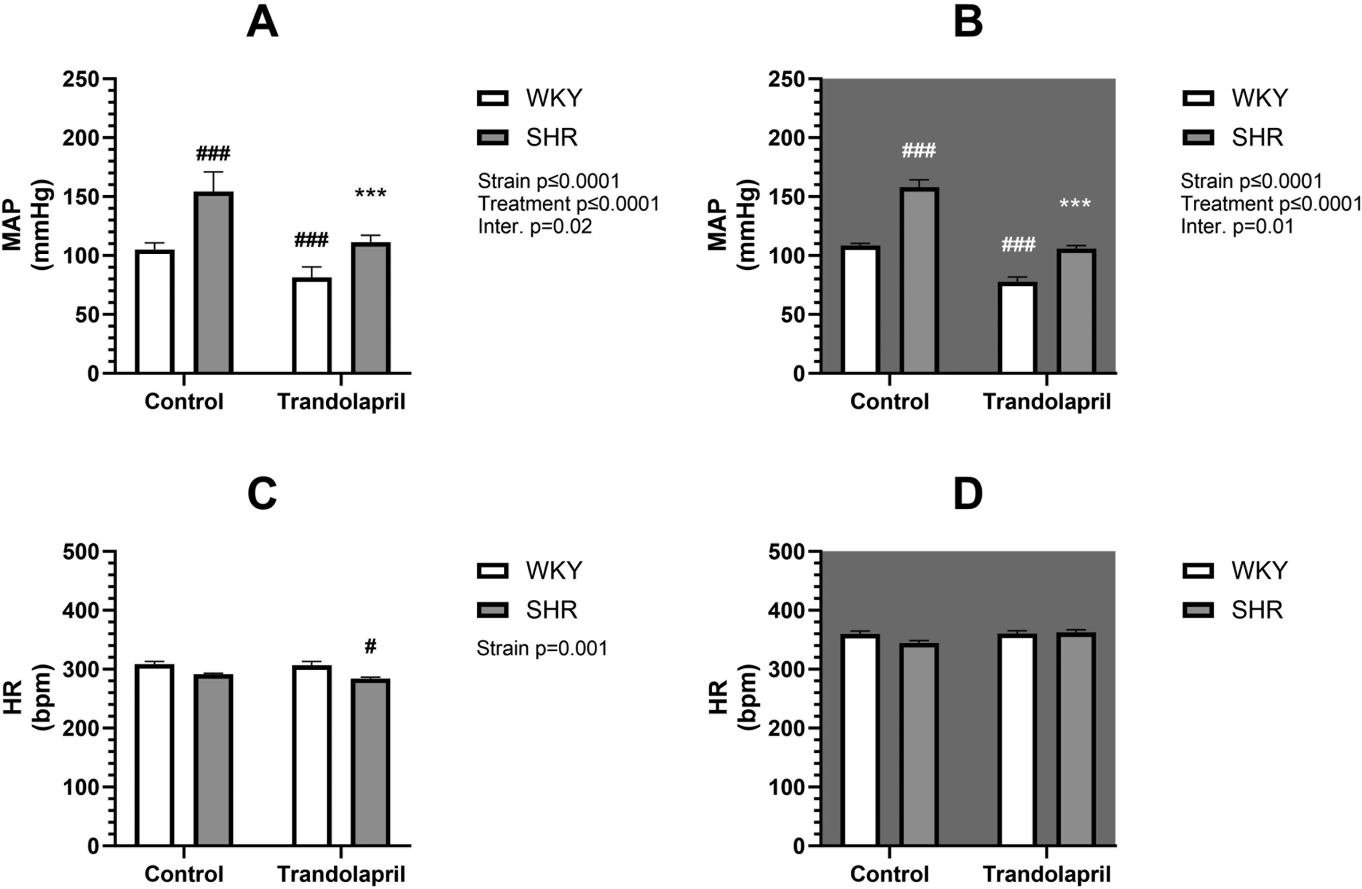
Arterial pressure and heart rate in SHR and WKY rats before and after treatment with TRA. Mean arterial pressure (MAP) **(A,B)** and heart rate (HR) **(C,D)** were recorded telemetrically during the light **(A,C)** and dark **(B,D)** phases (control: *n* = 8; PYR: *n* = 5). Data are expressed as mean ± SEM (*n* = 8 animals per group). *p ≤ 0.05 vs. corresponding control within the strain; ^#^*p* ≤ 0.05 vs. untreated WKY group. ***p ≤ 0.001 vs. corresponding control within the strain; ^#^*p* ≤ 0.05, ^###^*p* ≤ 0.001, vs. untreated WKY group.

Telemetric measurements were performed during 8 weeks of treatment with PYR or TRA. The mean arterial pressure (MAP) and HR data of each animal from the last 3 days and nights before treatment and from the last week of treatment were averaged and statistically compared. SHR control rats exhibited significantly higher (by approximately 60%) MAP than WKY control rats in both the light and dark phases of the day (Figures 1, 2, upper panels). SHR also showed a tendency towards a lower HR, compared to WKY, but only in the light phase (Figures 1, 2, lower panels). Eight weeks of PYR treatment slightly but significantly altered MAP in SHR during the dark phase by −8 ± 1 mmHg (Figure 1B). In terms of HR reduction, chronic PYR treatment in SHR reduced HR by −21 ± 3 bpm and −11 ± 3 bpm in both the light and dark phases, respectively (Figures 1C,D). Treatment with TRA significantly lowered MAP in both strains regardless of the day period (Figure 2). Trandolapril changed MAP by −24 ± 2 mmHg and −43 ± 6 mmHg in WKY and SHR, respectively, during the light period (Figure 2A), and by −31 ± 2 mmHg and −52 ± 7 mmHg in WKY and SHR, respectively, during the dark period (Figure 2B). HRs remained unchanged by TRA (Figures 2C,D). The changes in MAP induced by TRA were of greater magnitude compared to those induced by PYR treatment.

**Figure 3:**
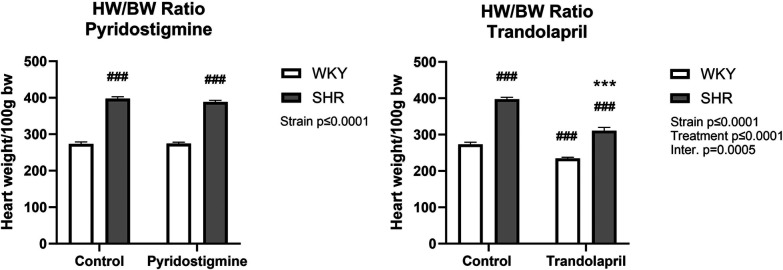
Heart weight in relation to body weight in SHR and WKY rats. Ratio of heart weight to body weight (HW/BW) after PYR treatment (left) or TRA treatment (right) was expressed as heart weight per 100 g body weight. Data are expressed as mean ± SEM (*n* = 8 animals per group). ***p ≤ 0.001 vs. corresponding control within the strain; ^###^*p* ≤ 0.001 vs. untreated WKY group.

As expected, a difference in heart weight to body weight ratio (HW/BW) was observed between SHR and WKY rats (Figure 3). Treatment with PYR did not affect this ratio in either strain (Figure 3; left), whereas treatment with TRA significantly reduced the HW/BW ratio in both strains, showing an antihypertrophic effect in SHR (Figure 3; right).

### 3.2 Baroreflex sensitivity and cardiac vagal tone

**Figure 4:**
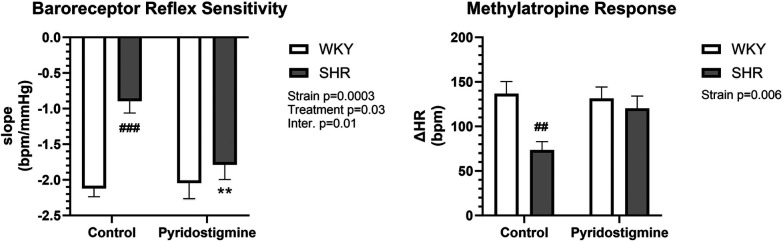
Baroreceptor reflex sensitivity and methylatropine HR response in SHR and WKY rats. Baroreceptor reflex sensitivity (left) and methylatropine HR response (right) were collected before and after treatment with PYR or TRA. Data are expressed as mean ± SEM (*n* = 8 animals per group). **p ≤ 0.01 vs. corresponding control within the strain; ^##^*p* ≤ 0.01 and ^###^*p* ≤ 0.001 vs. untreated WKY group.

Measurement of BRS expectedly showed a strain difference and significantly reduced BRS in control SHR (−0.89 ± 0.17) compared to control WKY (2.12 ± 0.12) (Figure 4; left). Furthermore, chronic PYR treatment significantly improved BRS in SHR to the level of control WKY, with slopes of −2.05 ± 0.22 and −1.79 ± 0.21 for treated WKY and SHR, respectively (Figure 4; left). Measurement of the methylatropine HR response showed a significantly reduced vagal or parasympathetic component of HR in control SHR compared to WKY (Figure 4; right). In SHR, chronic PYR treatment rescued the MA HR response to the untreated WKY level, i.e., increased vagal or parasympathetic tone (Figure 4; right).

### 3.3 Echocardiography

**Figure 5:**
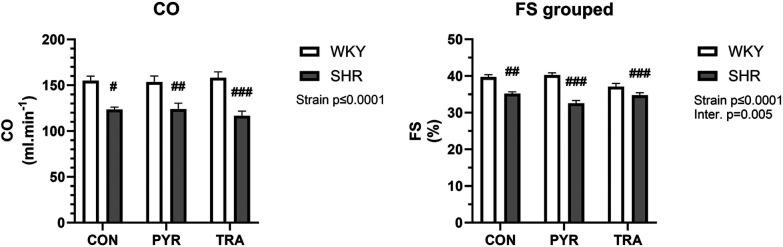
Echocardiographic data of cardiac output (CO) and fractional shortening (FS) before (CON) and after chronic pyridostigmine (PYR) treatment. Data are as mean ± SEM (*n* = 8–12 animals per group). ^#^*p* ≤ 0.05, ^##^*p* ≤ 0.01 and ^###^*p* ≤ 0.001 vs. untreated WKY group.

Echocardiographic measurements showed differences in diastolic anterior wall thickness (AWTd) and diastolic posterior wall thickness PWTd where both were significantly higher in SHRs corresponding with developed cardiac hypertrophy (Supplementary Table S1). Cardiac output (CO) and fractional shortening (FS) were smaller in SHRs than in WKY rats and both treatments induced no significant changes (Figure 5). PYR significantly increased only stroke volume (SV) in SHRs in comparison to untreated controls. PYR slightly reduced AWTd and anterior wall thickness (AWTs) in SHRs but this trend did not reach a significance. PYR enhanced the differences in systolic left ventricle diameter (LVDs) and HR between SHR and WKY rats where LVDs remained markedly higher, whereas HR was lower in SHRs (Supplementary Table S1). TRA was more effective than PYR and in WKY rats reduced AWTs and AWTd, systolic and diastolic left ventricle diameters (LVDs, LVDd), systolic posterior wall thickness (PWTs), and relative wall thickness (RWT) in comparison to untreated controls (Supplementary Table S1). In SHR, TRA significantly reduced AWTs and AWTd, PWTs, PWTd and RWT and increased SV (Supplementary Table S1). Early ventricular filling velocity was higher in all SHR groups than in the corresponding WKY groups, but the difference was not significant (Supplementary Table S2). Isovolumic relaxation time (IVRTm) was significantly higher in untreated SHR compared to WKY and this difference was improved by TRA but nor by PYR (Supplementary Table S2).

### 3.4 Cholinergic signaling

**Figure 6:**
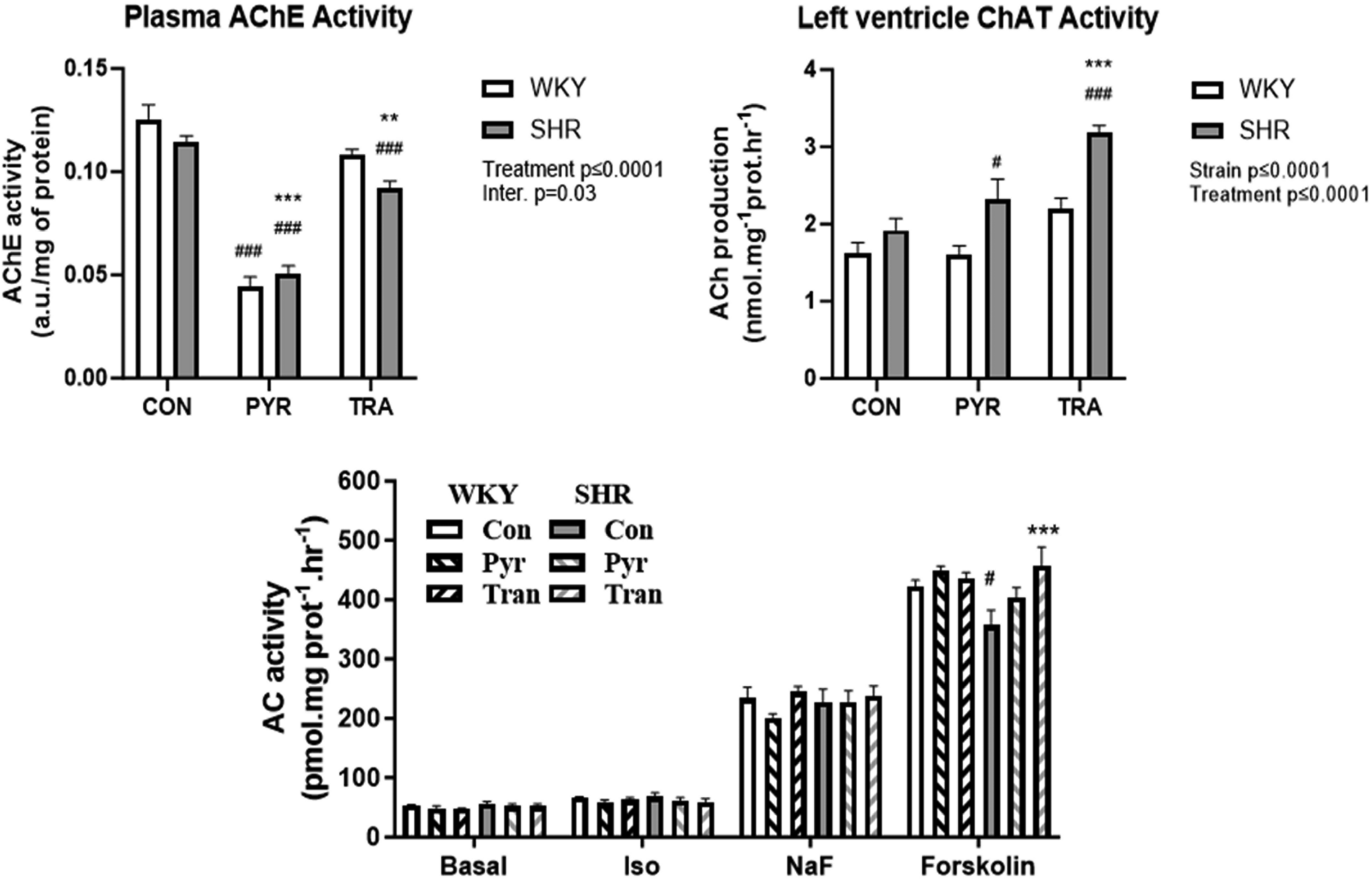
Acetylcholinesterase activity, choline acetyltransferase and adenylyl cyclase activity. AChE activity was determined in plasma, ChAT activity was determined in myocardial postnuclear supernatant, and AC activity was measured in crude myocardial membrane fractions from untreated (control) and PYR- or TRA-treated rats (E) Data are as mean ± SEM (*n* = 3). **p ≤ 0.01 and ***p ≤ 0.001 vs. corresponding control within the strain; ^#^*p* ≤ 0.05, ^###^*p* ≤ 0.001, vs. untreated WKY group.

We investigated the expression or function of selected components of cholinergic signaling in the left ventricles. We were interested in assessing the function of ChAT, the enzyme that catalyzes the formation of ACh. Therefore, the activity of ChAT was measured as the production of [^3^H]ACh. The measurement was performed on individual samples from the left ventricles of eight animals per experimental group (Figure 6, right). ACh production in the control groups of both rat strains was similar (WKY: 1.6 ± 0.4, SHR: 1.9 ± 0.4 nmol mg^−1^ protein hr^−1^). PYR and TRA had no significant effect on ChAT activity in WKY rats, but they increased the enzyme activity in SHR rats (by about 15% and 35%, respectively). The expression levels of ChAT and AChE did not differ significantly between all tested groups (Supplementary Figure S2).

Eight weeks of PYR treatment significantly reduced AChE activity in both strains by 65 ± 4% and 55 ± 3% from respective untreated control values in WKY and SHR, respectively. Chronic treatment with the ACE inhibitor TRA mildly but significantly reduced plasma AChE activity in both strains by approximately 14 ± 2% and 19 ± 3% in WKY and SHR, respectively (Figure 6, left).

Next, we were interested in determining the expression levels of β-AR and muscarinic receptors, selected G protein subunits and AC. We found no significant difference in the number of β-AR and muscarinic receptors, or in the amount of Gsα and Giα1,2 in the different preparations tested (Supplementary Figures S1, S2). Interestingly, the amount of Giα3 was lower in SHR than in WKY and treatment of SHR with PYR or TRA increased the expression of this protein to the level of WKY. There were no significant differences in the B_max_ and K_D_ values of muscarinic receptors in the myocardial preparations of SHR and WKA rats and between animals treated with PYR or TRA (Supplementary Figure S1).

The activity of AC was measured under basal and differently stimulated conditions (Figure 6, bottom panel). The β-AR signaling pathway of β-AR leading to AC activation was stimulated by the β-AR agonist isoprenaline, stimulation by NaF reflects the effect of G protein activation, and forskolin was used to measure maximally stimulated AC activity, because it activates both Gs protein and AC. Basal, isoprenaline- and NaF-stimulated activity did not differ in samples from both rat strains or between control and drug-treated animals. The values of basal, isoprenaline- and NaF-stimulated AC activity were in the range of 48–55 pmol.mg^−1^ protein hr^−1^, 58–68 pmol.mg^−1^ protein hr^−1^, and 201–245 pmol.mg^−1^ protein hr^−1^, respectively. Interestingly, AC activity stimulated by forskolin was lower in SHR rats (357 ± 44 pmol.mg^−1^ protein hr^−1^) than in WKY rats (and 423 ± 17 pmol.mg^−1^ protein hr^−1^) and trandolapril significantly increased this activity in SHR rats (457 ± 54 pmol.mg^−1^ protein hr^−1^).

### 3.5 Proteomic analysis of the pyridostigmine or trandolapril effects

To determine protein expression, six samples were measured by label-free LC-MS analysis. Samples were obtained from the left ventricles of control WKY rats (WC), WKY rats treated with PYR (WP) or TRA (WT), control SHR rats (SC) and SHR rats treated with pyridostigmine (SP) or trandolapril (ST). The intensities of triplicate samples were defined as median and then compared in seven pairwise comparisons (SC/WC, WP/WC, WT/WC, SP/SC, ST/SC, SP/WP and ST/WT). Proteins that showed qualitatively or quantitatively different expression levels between two experimental groups in a pairwise comparison were considered to be differently altered. A qualitative change was defined as the absence/presence of a protein in one experimental group in a pairwise comparison under the condition that the protein was detectable or undetectable in at least two out of three biological replicates. A quantitative change was defined as at least a twofold difference in protein expression level between two experimental groups and at the same time the protein was detected in at least in two out of three biological replicates.

**Figure 7:**
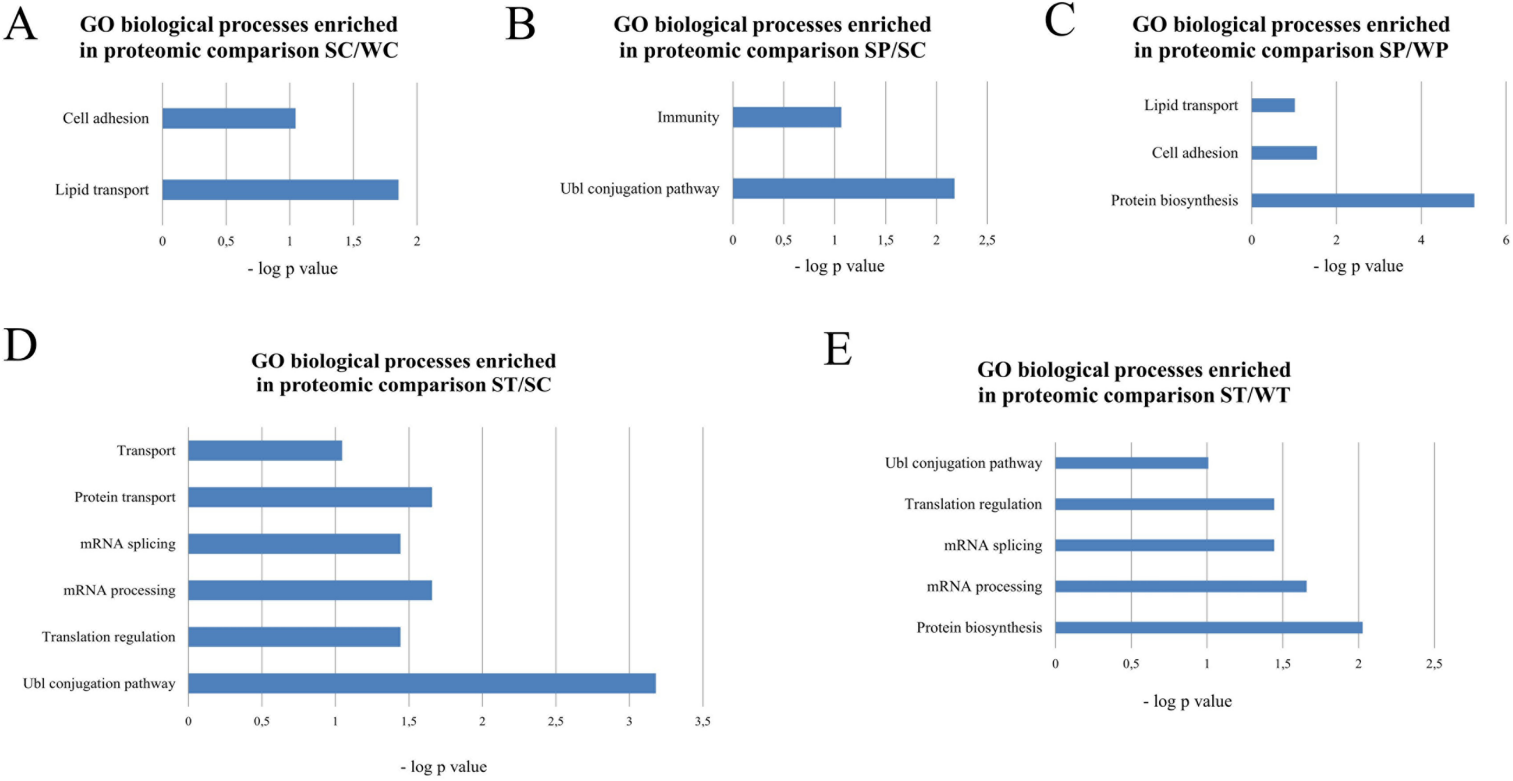
Gene ontology analysis of enriched biological processes by the DAVID tool. The *p*-values of enriched biological processes are indicated for proteomic comparisons SC/WC **(A)**, SP/SC **(B)**, SP/WP **(C)**, ST/SC **(D)** and ST/WT **(E)**.

GO analysis with the DAVID tool was performed for all pairwise comparisons. The control SHR and WKY control rats differed in the amount of proteins associated with cell adhesion and lipid transport (Figure 7A). No enriched GO biological processes were found in WKY rats after treatment with PYR or TRA. PYR treatment affected proteins related to the Ubl conjugation pathway or immunity in SHR rats (Figure 7B). The proteins that were differentially affected by PYR treatment in SHR and WKY rats were related to lipid transport, cell adhesion and protein biosynthesis (Figure 7C). The GO biological processes that were enriched in SHR rats treated with TRA included transport, mRNA splicing and processing and protein turnover (translational regulation and Ubl conjugation pathway) (Figure 7D). TRA had a significant effect on proteins related to protein turnover (protein biosynthesis, translation regulation and Ubl conjugation pathway) and mRNA splicing and processing (Figure 7E). Overall, TRA appears a more efficient drug compared to PYR. It has a greater impact on protein turnover and even affects mRNA processes but only in SHR rats. A further detailed analysis, including a more comprehensive GO analysis and other protein-protein associations, is described in Supplementary Results and shown in Supplementary Figures S3–S7. Here, we present the important protein changes associated with protein turnover and mRNA processes.

**Figure 8:**
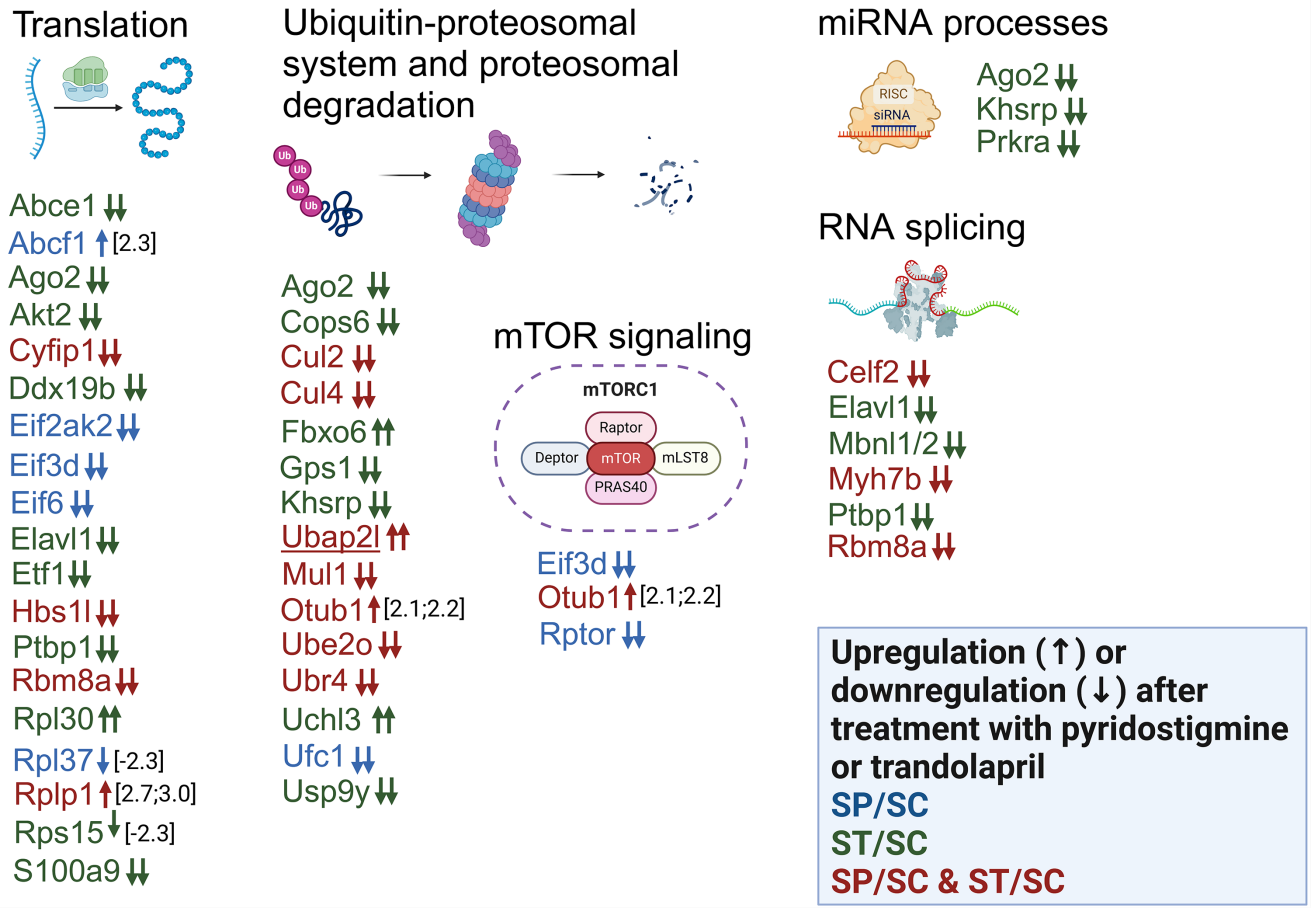
Alterations in the expression of proteins involved in biological processes related to protein turnover, mRNA processes and mTOR signaling. Differentially expressed proteins after administration of PYR or TRA to SHR rats are sorted according to the gene ontology analysis provided by the DAVID tool. The effect of PYR or TRA alone on the expression of certain proteins is shown in blue or green color. The simultaneous effect of both inhibitors on protein expression is shown in red color. Small upward and downward arrows represent an increase and a decrease in expression, respectively. Two or one arrow stand for qualitative or quantitative changes. The gene names of the proteins with different expression between control WKY and SHR rats are underlined.

The inhibitors administered to SHR rats also led to changes in the levels of seventeen proteins related to cytoplasmic translation (Abce1, Abcf1, Ago2, Akt2, Cyfip1, Eif2ak2, Eif3d, Eif6, Elavl1, Etf1, Hbs1L, Ptbp1, Rbm8a, Rpl37, Rplp1, Rps15 and S100a9) (Figure 8; Supplementary Table S4). In SHR rats, PYR induced loss of the initiation factors Eif3d and Eif6 (Figure 8; Supplementary Table S4). Loss of Abce1 or Hbs1L was induced by TRA or both inhibitors in SHR rats (Figure 8; Supplementary Table S4). The level of Pelo was undetectable in SHR control rats compared to WKY control rats and both inhibitors had no effect on its expression (Supplementary Table S5). The proteomic profile of mTOR scaffold protein Rptor (Raptor) in SHR rats was the same as that of Eif3d and Eif6 (Figure 8; Supplementary Table S4). It suggests that PYR may regulate mTOR signaling and S6K1 phosphorylation with subsequent effects on the assembly of the eIF3 initiation complex. Cyfip1 levels were undetectable in SHR rats receiving PYR or TRA compared to SHR control rats (Figure 8; Supplementary Table S4).

The eukaryotic release factor Etf1 (eRF1) and the DEAD box RNA helicase Ddx19b were not detectable in SHR rats administered TRA compared to SHR control rats (Figure 8; Supplementary Table S4), suggesting that TRA may suppress translation termination. Alterations in the levels of four ribosomal proteins may be related to assembly and maturation of the 60S and 40S ribosomal subunits regulated by Eif6. Rplp1, Rpl30 and Rpl37a or Rps15 are components of large (60S) or small (40S) ribosomal subunits. The levels of these proteins were differentially altered by PYR or TRA (Figure 8; Supplementary Table S4), suggesting that both inhibitors may affect ribosomal subunit assembly.

TRA was found to affect the levels of three proteins (Ago2, Khsrp and Prkra) related to the miRNA metabolic process and the loading of miRNA to the RISC complex (Figure 8; Supplementary Table S4). The levels of Ago2, Khsrp and Prkra were undetectable in SHR rats administered TRA (Figure 8; Supplementary Table S4). This process could be induced by TRA in SHR rats. Some proteins associated with proteasomal degradation (Fbxo6, Cul2, Cul4b) were also altered in different ways (Figure 8; Supplementary Table S4). In contrast to SHR control rats, Fbxo6 was detected in SHR rats administered TRA (Figure 8; Supplementary Table S4), but Cullin 2 (Cul2) and cullin 4B (Cul4b) were undetectable in SHR rats treated with any of the inhibitors (Figure 8; Supplementary Table S4). The subunits of COP9 signalosome complex (CSN), Cops6 and Gps1, were undetectable in SHR rats administered TRA (Figure 8; Supplementary Table S4). These data indicate that TRA may affect the ubiquitin-proteasomal system by reducing the subunit levels of cullin-RING ligases and the COP9 signalosome complex.

The other differentially expressed proteins (Otub1, Ubr4, Ube2o, Ufc1, Uchl3, Usp9y and Mul1) are associated with the ubiquitin-proteasome system (Figure 8; Supplementary Table S4). The level of the ubiquitin thioesterase, Otub1, was slightly increased in SHR rats receiving PYR or TRA (Figure 8; Supplementary Table S4). The level of the ubiquitin carboxy-terminal hydrolase isozyme L3, Uchl3, was detectable only in SHR rats treated with TRA (Figure 8; Supplementary Table S4). The levels of the other proteins were suppressed by PYR or TRA (Figure 8; Supplementary Table S4). Several differentially expressed RNA-binding proteins have been found to be associated with RNA splicing. Ptbp1 and Mbnl1/2 regulate alternative splicing in the heart. TRA treatment made both Ptbp1 and Mbnl1/2 undetectable in SHR rats (Figure 8; Supplementary Table S4).

## 4 Discussion

The main physiological findings of the present study are that PYR can slightly decrease MAP and HR and greatly improve BRS in SHR, and that this improvement is achieved by potentiating parasympathetic tone. Treatment with TRA was very effective in lowering MAP and cardiac hypertrophy in SHR. MAP was increased in SHR compared to WKY during both phases of the day, as expected, and treatment with PYR slightly but significantly reduced MAP during the dark phase. On average, HR was slightly lower in SHR than in WKY during the light rest phase and similar during the dark active phase. PYR showed a tendency to reduce HR in both strains, regardless of the time of day, but this effect was more pronounced during the light phase. These findings are in accordance with those of Blanco et al. (20), where SHR and WKY rats were treated with the same dose of PYR (25 mg/kg/day) for 2 weeks. TRA lowered MAP more effectively in both strains during both phases of the day and had no effect on HR in either strain. This is consistent with the results of Baillard et al. (39), who found that TRA at the same dose and treatment regimen reduced blood pressure in SHR and Wistar rats.

Plasma AChE activities were similar in both rat strains. Treatment with PYR strongly reduced AChE activity to a similar extent in both strains. Interestingly, TRA was able to slightly but significantly reduce AChE activity in plasma of both SHR and WKY, although it has no direct AChE inhibitory effect. This finding could be explained by the fact that BRS resets to lower blood pressure levels due to the strong influence of TRA on MAP, possibly reducing parasympathetic activity. This resetting could consequently reduce ACh production and thus AChE activity in plasma. Importantly, BRS resetting has been proposed as one of the mechanisms of action of ACE inhibitors (19, 40, 41). In parallel, local myocardial ChAT activity did not differ between SHR and WKY rats and was significantly increased by TRA only in SHR. As expected, BRS was attenuated in SHR compared to WKY, and treatment with PYR successfully raised BRS to the level of normotensive WKY controls. This was achieved by an increase in parasympathetic tone, as can be seen from the MA HR response, where PYR treatment increased this response of SHR to that of normotensive WKY controls. This result is consistent with the findings of Blanco et al. (20).

As expected, relative heart weights indicated developed cardiac hypertrophy in SHR rats. In contrast to TRA, PYR did not reduce relative heart weights and therefore did not reduce cardiac hypertrophy in SHR. Interestingly, Liu et al. (42) reported that chronic i.p. administration of choline increased parasympathetic tone, lowered blood pressure and HR, improved cardiac function and BRS, and could reduce the relative heart weight of SHR, a finding suggesting that elevated ACh levels could alleviate cardiac hypertrophy. To our knowledge, other authors have not found antihypertrophic effects of PYR. On the other hand, the antihypertrophic effects of TRA are well documented and are also consistent with our proteomic results discussed below (14, 15, 39).

Echocardiographic examination revealed differences between the strains in alignment with other acquired data in our study but found no major effect of either PYR or TRA on cardiac output or fractional shortening. Interestingly, Gardim et al. (22) investigated the effects of PYR treatment with 15 mg/kg/day on hemodynamic parameters of SHR and found increased cardiac output associated with treatment, an opposite result to ours, but they normalized other parameters to animal body weight without providing body weight data, which prevents further comparisons. Baillard et al. (39) examined the effects of TRA on cardiac hemodynamic parameters in older, middle age (12–14-month-old) SHRs using the same treatment regimen as ours and found similar results to ours where heart diameters were reduced by TRA, yet they reported no changes in cardiac output or fractional shortening. These and our results point exclusively to antihypertrophic effects of TRA without affecting cardiac function.

We could not detect any significant differences between the number of myocardial β-AR and muscarinic receptors or in the expression of selected G protein subunits, key components of the AC signaling system, in the two rat strains tested. They were neither influenced by PYR nor by TRA, except for Giα3. The differentially stimulated AC activity was mostly not different in the samples from rats treated with both drugs tested. The only exception was AC activity stimulated by forskolin, which was lower in SHR rats than in WKY rats and normalized by TRA. This could possibly be explained by a lower expression of myocardial AC in SHR rats due to a repetitive activation by catecholamine-agonized β-adrenergic receptors, which could be restored by the sympatholytic effect of TRA.

Our results of proteomic analysis clearly show that the myocardial proteome profiles of WKY and SHR rats differ significantly, and that inhibition of acetylcholinesterase or angiotensin-converting enzyme markedly affects protein expression depending on the phenotype of the animal strains. This means that the proteomic profile underlying the effects of antihypertensive drugs should be considered when studying hypertension. Some differences in protein expression observed in SHR control rats compared with WKY control rats were restored to the level of WKY control rats by PYR or TRA treatment (Supplementary Table S3), but many protein alterations were unaffected by these inhibitors. Both inhibitors affected the expression of many proteins that did not differ between WKY and SHR control rats. Overall, this raises the question of whether WKY rats can be used as a normotensive control for studying drug effects on hypertension in SHR rats.

Proteomic analysis of the effects of PYR and TRA in the left ventricles of rat hearts revealed that both inhibitors significantly affect expression the levels of a number of proteins. TRA, a drug reducing the formation of angiotensin II (43, 44), was found to be more efficient in regulating protein expression. Both inhibitors apparently control the expression of many proteins in same way. The overlap of their effects on protein expression could be explained by the interaction between the renin-angiotensin system and the cholinergic system. The association of both systems has been reported to influence cardiac functions such as blood pressure or heart rate (45–47). It appears that these systems are linked at the level of regulation of protein expression, resulting in different proteomic profiles that contribute to cardiovascular phenotypes. To date, no study has been conducted on the effect of acetylcholine on the cardiac proteome. Only a few studies have addressed proteomic changes induced by ACE inhibitors (48, 49).

Proteolysis, which is involved in protein degradation, is one of the biological processes enriched in the transcriptome of the left ventricles of SHR rats with heart failure (50). The proteins that form the proteasome regulatory particles were differentially expressed in the renal arteries of SHR rats compared to WKY rats (51). Activated protein turnover, which relates to protein synthesis and degradation, contributes to left ventricular hypertrophy due to increased cardiac workload (52). The ubiquitin-proteasome, which ensures protein degradation, is also a key component of the quality control mechanism in protein synthesis (53). Increased protein synthesis by cardiac myocytes is required for cardiac hypertrophy, but is accompanied by an increase in the cellular amount of misfolded and aberrant proteins (53, 54). The upregulation of proteasome expression and activity balances the cellular accumulation of *de novo* synthesized proteins (52). Our analysis revealed that PYR and TRA directly affect the expression of proteins involved in protein synthesis and degradation, while forming the distinct proteome profiles in the left ventricle. The altered proteins include the members and enzymes of the ubiquitin conjugation pathway, which is responsible for the formation and regulation of the E3 ubiquitin ligase complexes. At the same time, both inhibitors triggered far-reaching alterations leading to different proteome profiles of proteins involved in several steps of protein synthesis, including mRNA splicing and metabolism, miRNA processes and translation.

Our data suggest that both the cholinergic system and the renin-angiotensin system regulate mTOR signaling (Figure 8). Little is known about the relationship between acetylcholine and this signaling pathway. Deletion of Raptor resulted in selective disruption of mTORC1 signaling and alterations in vascular function in endothelial or smooth muscle cells, as week as reduced acetylcholine-induced relaxation responses in the aorta (55). The effect of angiotensin II on mTOR signaling in the cardiovascular system has been better studied (55–58). Our results provide evidence that inhibition of acetylcholine breakdown or angiotensin I to angiotensin II conversion directly affects mTOR signaling by modulating proteome profiles of these pathways.

The mTOR signaling pathway is involved in cardiac hypertrophy (59, 60). The mTORC1 complex with the interacting protein Raptor (Rptor) is a regulatory site for protein synthesis (60, 61). In our study, Raptor was found to be in SHR and WKY rats treated with PYR, in contrast to controls, while TRA had no effect on Raptor expression. Deletion of Raptor in C57BL/6J mice reduced protein synthesis and led to thickening of the left ventricular posterior wall and increased left ventricular internal diameter (62). This suggests that PYR would suppress protein synthesis and decrease heart weight including left ventricular parameters in both strains, but treatment with PYR did not cause changes in heart weight. In contrast, treatment with TRA reduced heart weight in both strains, indicating a relationship between Raptor expression and heart weight. The relationship between TRA and the reduction of cardiac hypertrophy is also supported by our finding that the expression of Mbnl1 and Ptpb1 was reduced in TRA-treated SHR rats. Although our data are in contrast with previous findings (62), they support the notion that Raptor is an important regulatory phosphoprotein whose cellular levels and post-translational modifications determine the activation or extent of protein synthesis.

## 5 Conclusion

Both PYR and TRA are able to lower BP in SHR rats. PYR, unlike TRA, can slightly decrease HR and effectively increase BRS through vagal potentiation. The myocardial proteome profiles of proteins involved in protein synthesis and degradation differ greatly between SHR and WKY rats, and specific differences were also observed between the effects of PYR and TRA. Only Cops6, a protein involved in protein degradation, appears to be the only potential hypertrophic biomarker whose expression correlates with heart weight in both treatments with the inhibitors and in both strains. This suggests that alterations in the expression of proteins involved in protein turnover may contribute to a greater effect of TRA on cardiac hypertrophy compared to PYR.

## Data Availability

The mass spectrometry proteomics data have been deposited to the ProteomeXchange Consortium via the PRIDE partner repository under the accession number PXD050397.
