# Supplementary material for: Modulation of left ventricular hypertrophy in spontaneously hypertensive rats by acetylcholinesterase and ACE inhibitors: physiological, biochemical, and proteomic studies
Source: Front Cardiovasc Med. 2024 Sep 16;11:1390547. doi: 10.3389/fcvm.2024.1390547 (PMC11443425; doi:10.3389/fcvm.2024.1390547)
Supplement: Supplementary file 2 [file Datasheet1.pdf]

Supplementary Material

Supplementary Figures

A

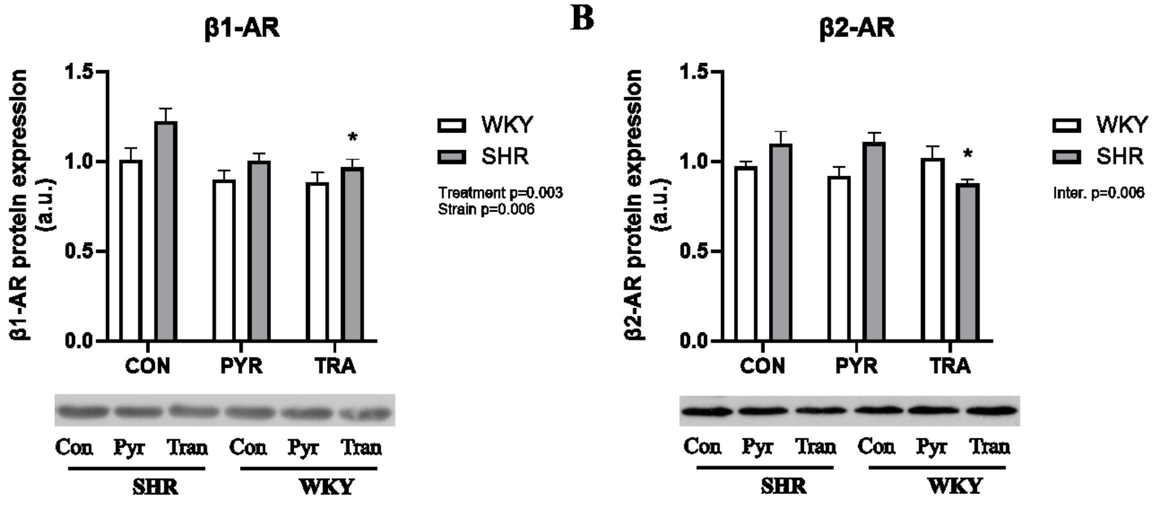

B

C

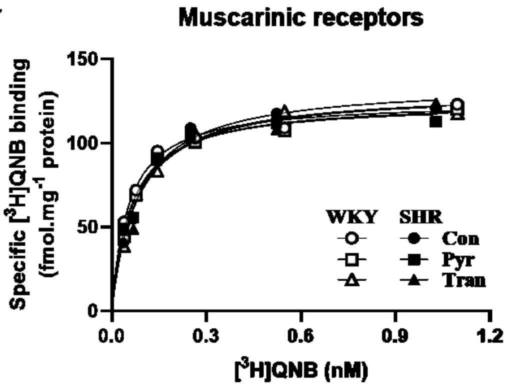

D

		K_D (pM)	B_{max} ($fmol.mg^{-1}$)
WKY	CON	67.3 ± 0.8	132 ± 6
	PYR	66.1 ± 0.1	122 ± 3
	TRA	58.9 ± 1.0	110 ± 11
SHR	CON	95.6 ± 11.9	133 ± 5
	PYR	66.5 ± 0.3	118 ± 11
	TRA	64.4 ± 0.9	112 ± 11

Supplementary Figure S1. Abundances of β-adrenergic receptors (β-AR) and muscarinic receptors in left ventricular tissue. The expression of β1-AR (A) and β2-AR (B) was assessed by Western blotting. Muscarinic receptors were detected using the radioligand binding method. Representative binding curves (C) and binding parameters (D) are shown. Data expressed as mean ± SEM (n = 6). * $p \leq 0.05$ vs control within the strain.

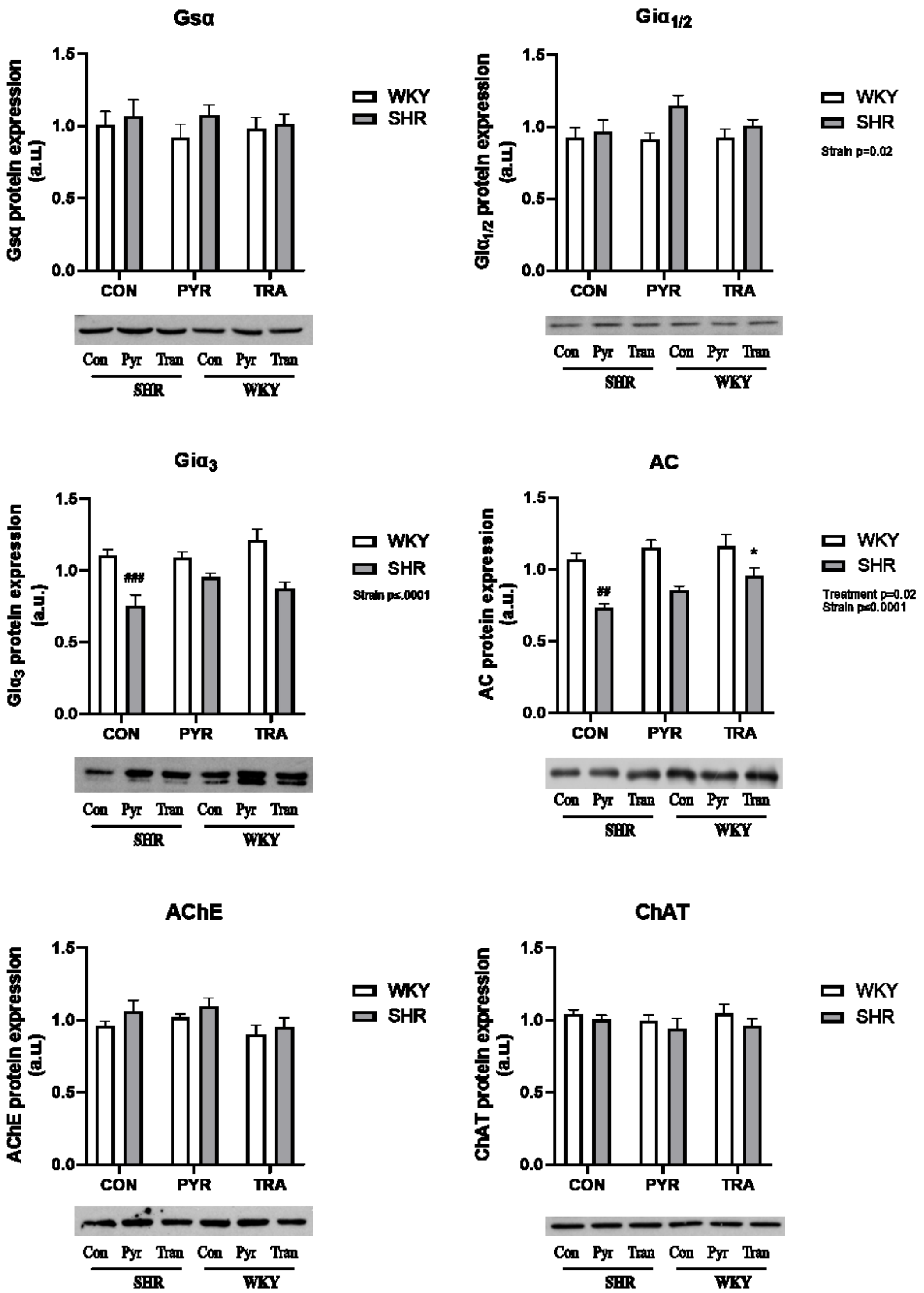

Supplementary Figure S2. Abundances of G proteins, adenylyl cyclase (AC), acetylcholinesterase (AChE) and choline acetyltransferase (ChAT) in left ventricular tissue as assessed by Western blotting. Data expressed as mean ± SEM (n = 6). * $p \leq 0.05$ vs control within the strain; $^{\#}$ $p \leq 0.05$ vs. WKY control.

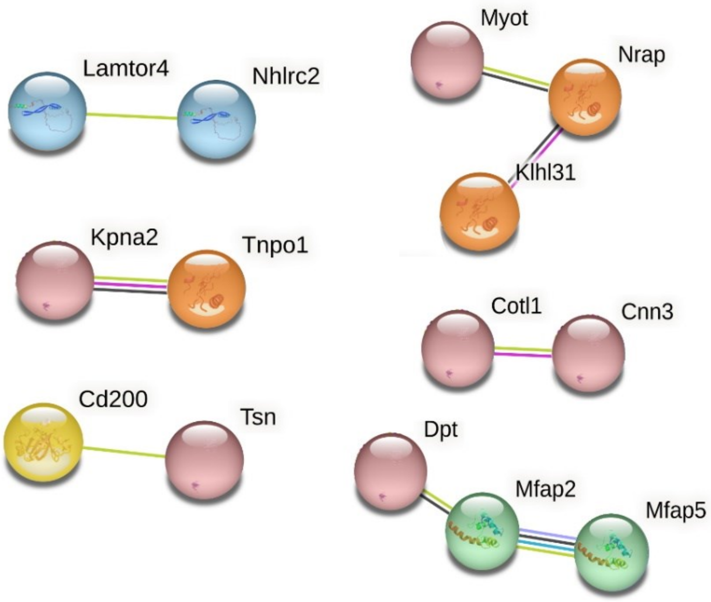

Supplementary Figure S3. Network representation of differentially expressed proteins in the rat left ventricles of WKY or SHR control rats and WKY or SHR rats treated with pyridostigmine or trandolapril. Thirty-one differentially expressed proteins were mapped onto the rat STRING database and connected in a tight network with 36 edges. The green and red nodes represent proteins with increased and decreased levels, respectively, in samples from SHR rats treated with pyridostigmine or trandolapril compared to SHR control rats. The light blue or yellow nodes represent proteins with increased and decreased levels, respectively, in the left ventricles of SHR rats administered only pyridostigmine relative to control SHR rats. The dark blue and orange nodes represent proteins with increased and decreased levels, respectively, in the left ventricles of SHR rats administered trandolapril alone compared to control SHR rats. The average node degree is 1.26 and the enrichment p-value of protein-protein interactions is 1.17e-07.

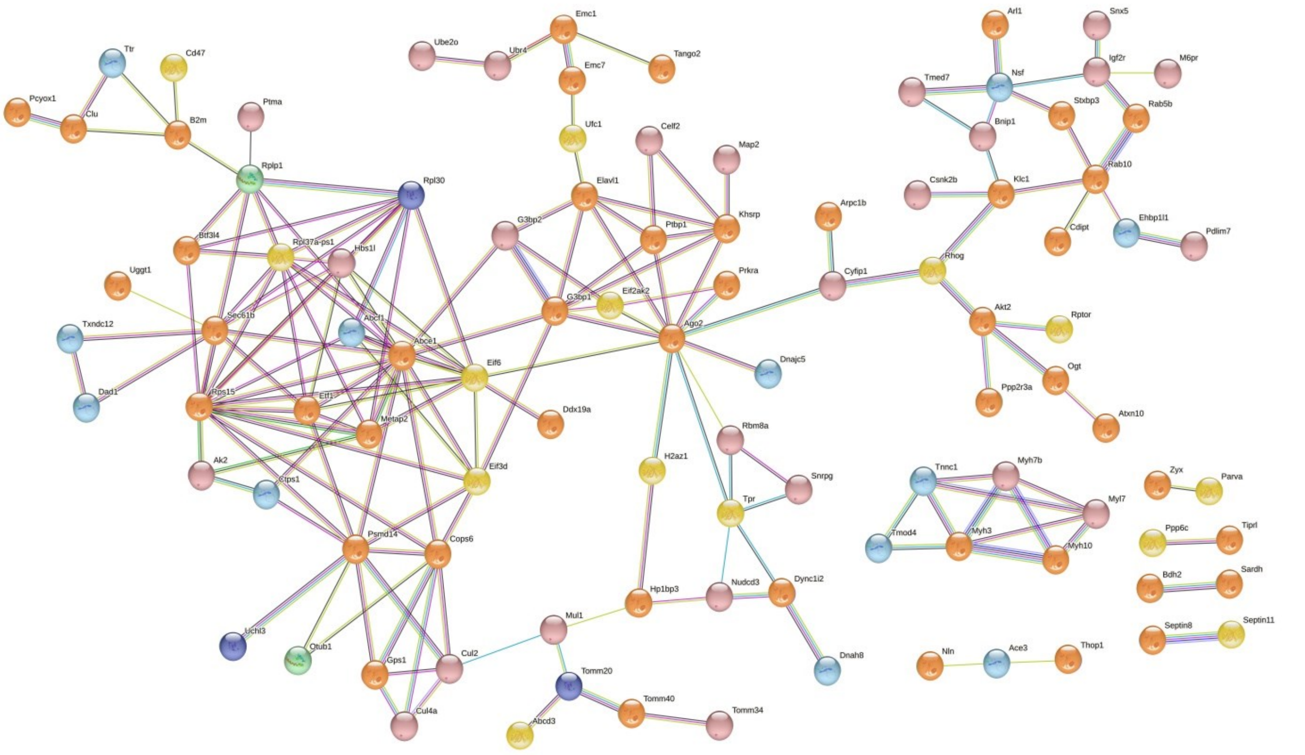

Supplementary Figure S4. Network representation of differentially expressed proteins in rat left ventricles from control WKY or SHR rats and WKY or SHR rats treated with pyridostigmine or trandolapril. One hundred fifty-nine differentially expressed proteins were mapped onto rat STRING database and connected in a tight network with 370 edges. The green and red nodes represent proteins with increased and decreased levels, respectively, in samples from SHR rats treated with pyridostigmine or trandolapril compared to SHR control rats. The light blue or yellow nodes represent proteins with increased and decreased levels, respectively, in the left ventricles of SHR rats administered only pyridostigmine compared to SHR control rats. The dark blue and orange nodes represent proteins with increased and decreased levels, respectively in the left ventricles of SHR rats administered only trandolapril compared to SHR control rats. The average node degree is 3.92 and the enrichment p-value of protein-protein interactions is 4.56e-12.

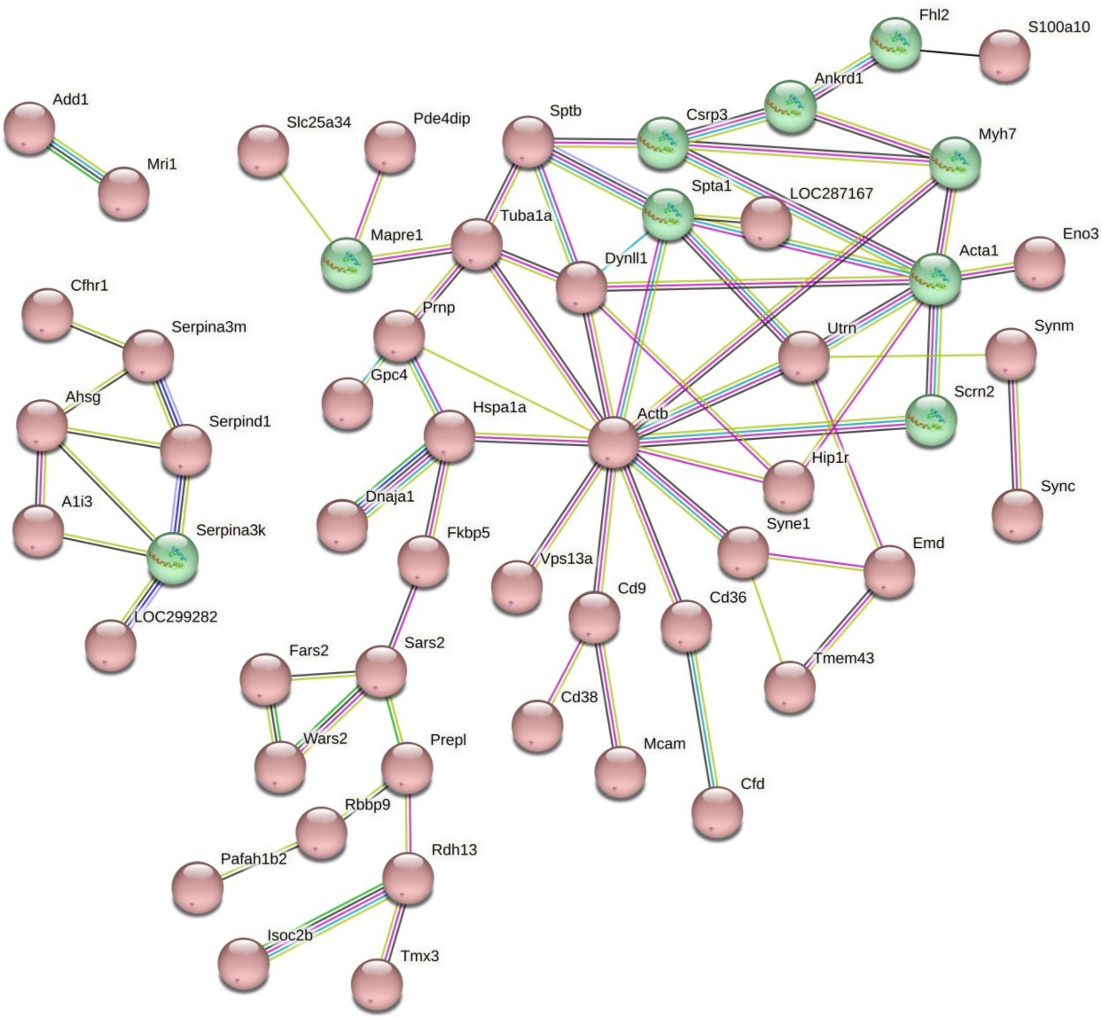

Supplementary Figure S5. Network representation of differentially expressed proteins in the left ventricles from of WKY or SHR control rats. Eighty-seven differentially expressed proteins were mapped onto the rat STRING database and connected in a tight network with 117 edges. The green and red nodes represent proteins with increased and decreased levels, respectively, in the left ventricles of SHR control rats compared to WKY control rats. The average node degree is 1.97 and the enrichment p-value of protein-protein interactions is 4.64e-08.

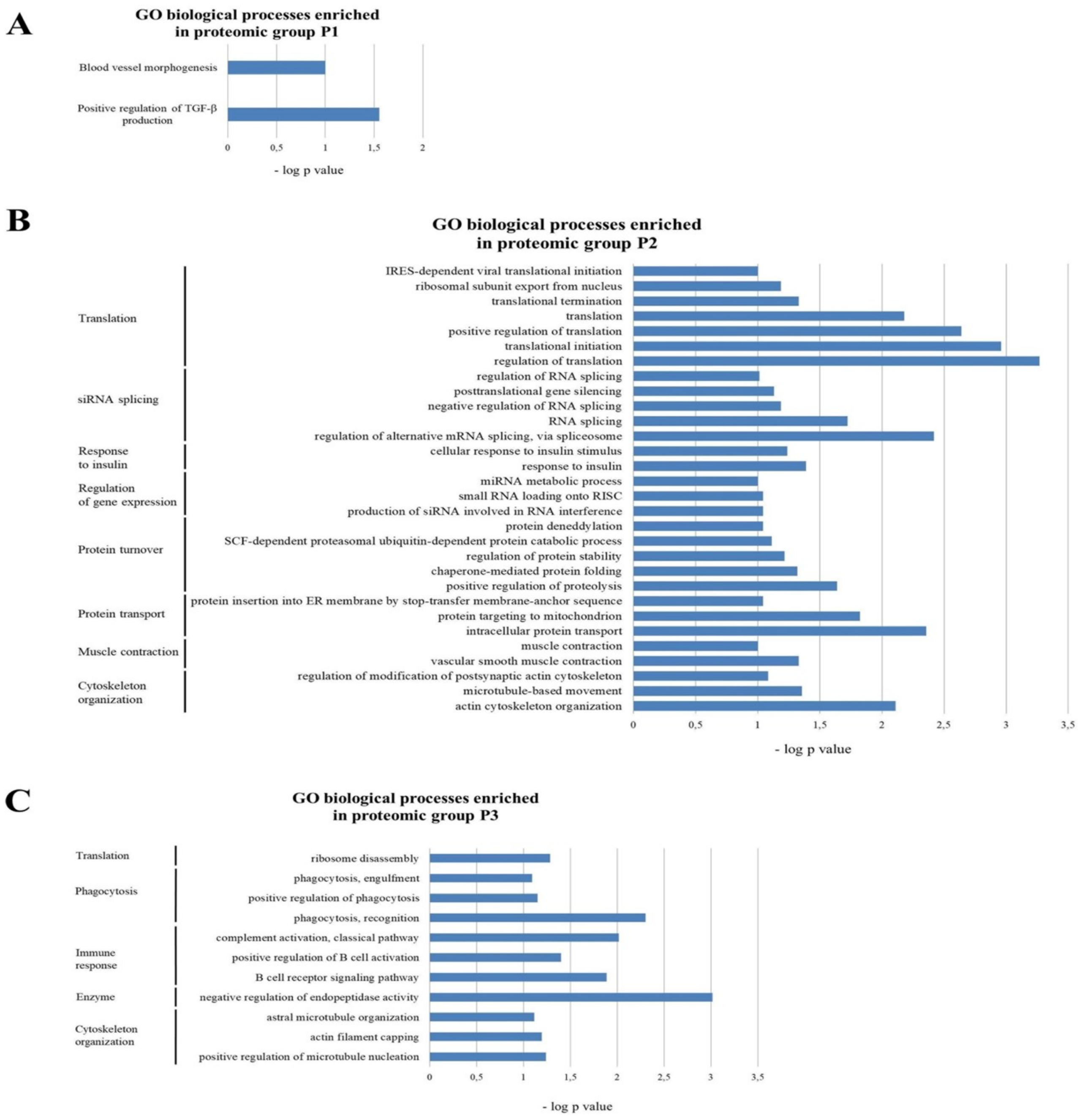

Supplementary Figure S6. Gene ontology analysis of enriched biological processes by the DAVID tool. The p-values of enriched biological processes are indicated for proteomic groups P1 (A), P2 (B) and P3 (C).

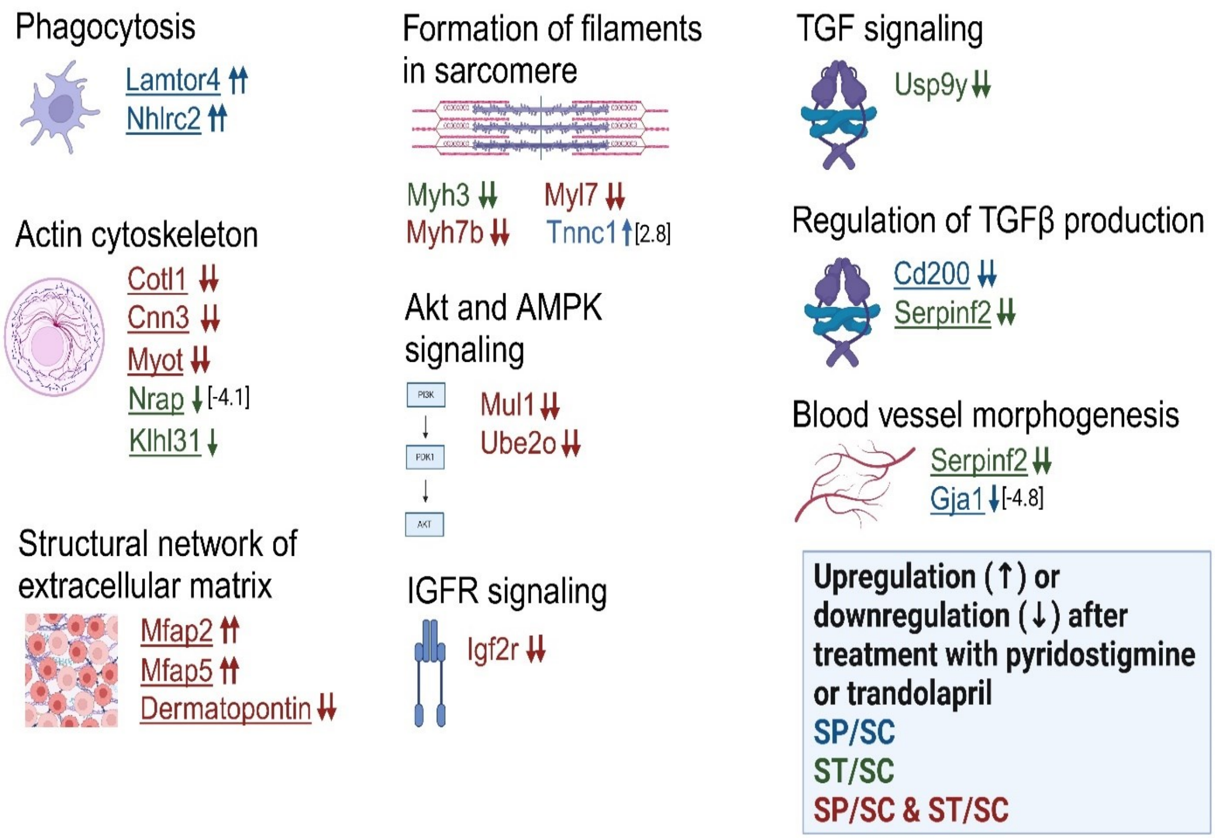

Supplementary Figure S7. Alterations in the expression of proteins involved in biological processes related phagocytosis, structural processes, signaling pathways and blood vessel morphogenesis. The effect of PYR or TRA alone on the expression of certain proteins is shown in blue or green color. The simultaneous effect of both inhibitors on protein expression is shown in red color. Small upward and downward arrows represent an increase and a decrease in expression, respectively. Two or one arrow represents qualitative or quantitative changes, respectively. The gene names of the proteins with different expression between control WKY and SHR rats are underlined.

Abbreviations: Cd200, OX-2 membrane glycoprotein; Cnn3, Calponin-3; Cotl1, Coactosin-like protein; Igf2r, Insulin-like growth factor 2 receptor; Gja1, Gap junction alpha-1 protein; Klhl31, Kelch-like family member 31; Lamtor4, Ragulator complex protein Lamtor4; Mfap2, Microfibril-associated protein 2; Mfap5, Microfibril-associated protein 5; Myh3, Myosin-3; Myh7b, Myosin heavy chain 7B; Myl7, Myosin light chain 7; Mul1, RING-type E3 ubiquitin transferase; Myot, Myotilin; Nhlrc2, NHL repeat containing 2; Nrap, Nebulin-related-anchoring protein; Serpinf2, Serpin family F member 2; Tnnc1, Troponin C1 slow skeletal and cardiac type; Ube2o, Ubiquitin-conjugating enzyme E2O; Usp9y, Ubiquitinyl hydrolase 1.

β1-AR β2-AR

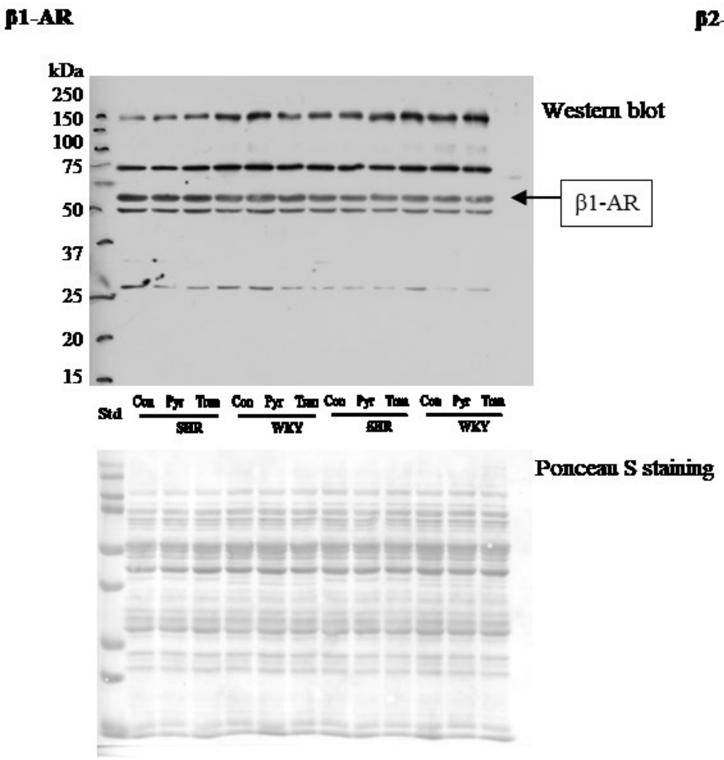

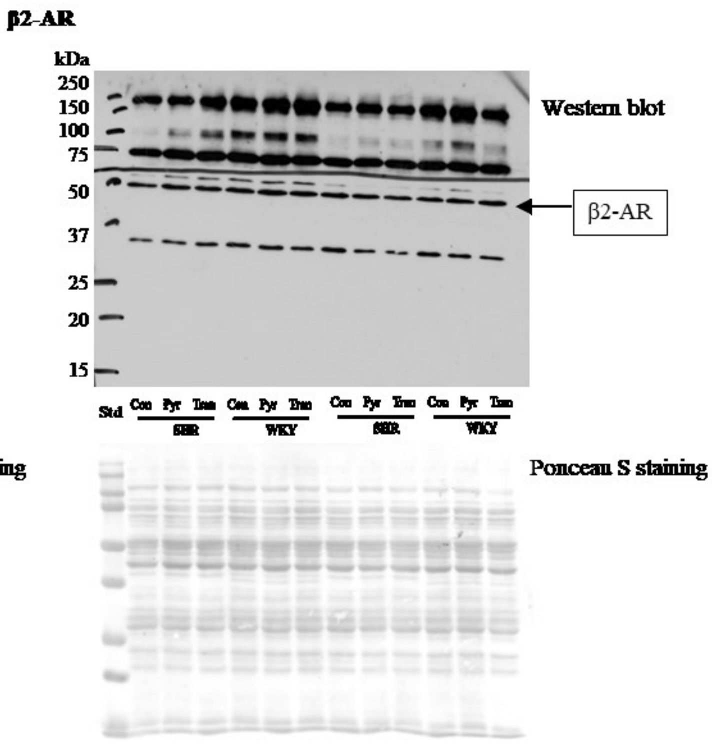

Supplementary Figure S8. Images of whole uncropped original Western blots and Ponceau S staining used as a loading control for the assessment of β1-AR and β2-AR, as shown in Suppl. Figure S1.

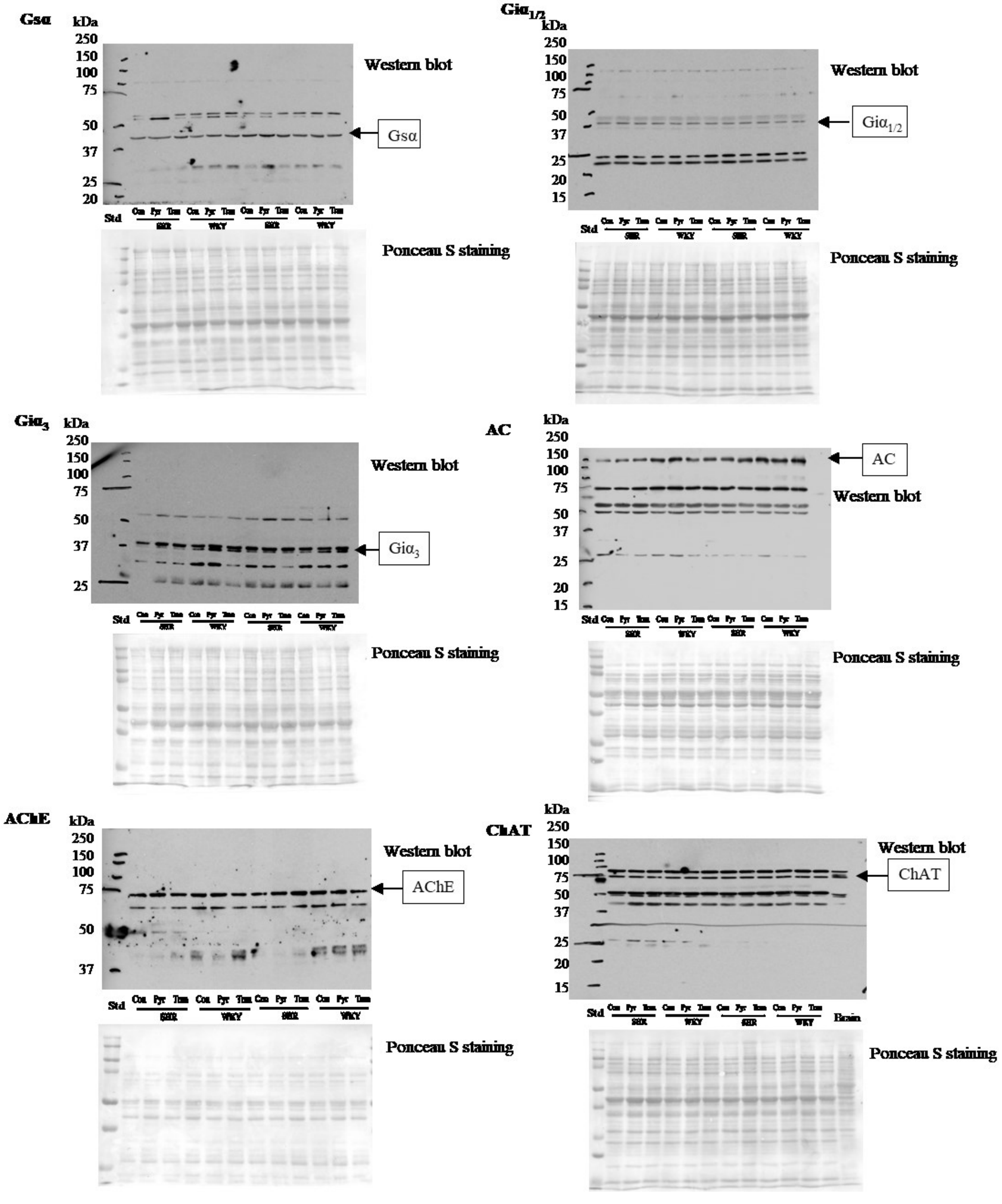

Supplementary Figure S9. Images of whole uncropped original Western blots and Ponceau S staining used as a loading control for the assessment of G proteins (Gsα, Giα$_{1/2}$, Giα$_{3}$), adenylyl cyclase (AC), acetylcholinesterase (AChE) and choline acetyltransferase (ChAT), as shown in Suppl. Figure S2.
