# Supplementary material for: Modulation of left ventricular hypertrophy in spontaneously hypertensive rats by acetylcholinesterase and ACE inhibitors: physiological, biochemical, and proteomic studies
Source: Front Cardiovasc Med. 2024 Sep 16;11:1390547. doi: 10.3389/fcvm.2024.1390547 (PMC11443425; doi:10.3389/fcvm.2024.1390547)
Supplement: Supplementary file 3 [file Datasheet2.pdf]

Supplementary Material

Supplementary Tables

Table S1. Echocardiographic parameters of left ventricle (LV) and heart rate (HR)

	Controls		Pyridostigmine		Trandolapril	
	**WKY**	**SHR**	**WKY**	**SHR**	**WKY**	**SHR**
AWTd (mm)	1.86 ± 0.03	2.17 ± 0.03#	1.85 ± 0.03	1.98 ± 0.05	1.64 ± 0.02#	1.81 ± 0.04*
LVDd (mm)	7.90 ± 0.1	7.73 ± 0.06	7.74 ± 0.08	7.96 ± 0.17	8.39 ± 0.15#	8.23 ± 0.09
PWTd (mm)	1.85 ± 0.04	2.07 ± 0.03#	1.88 ± 0.05	2.04 ± 0.06	1.69 ± 0.04	1.66 ± 0.03*
AWTs (mm)	2.75 ± 0.05	2.94 ± 0.08	2.72 ± 0.05	2.66 ± 0.06	2.48 ± 0.06#	2.57 ± 0.04*
LVDs (mm)	4.82 ± 0.09	5.08 ± 0.08	4.61 ± 0.07	5.42 ± 0.17†	5.36 ± 0.14#	5.14 ± 0.09
PWTs (mm)	2.72 ± 0.04	2.93 ± 0.02	2.81 ± 0.05	2.92 ± 0.09	2.43 ± 0.05#	2.37 ± 0.03*
RWT (%)	47.5 ± 1.5	54.8 ± 1.0#	48.3 ± 1.2	50.8 ± 2.0	39.7 ± 1.2#	44.8 ± 1.1*
SV (µl)	415.8 ± 15.6	344.8 ± 6.3	381.9 ± 14.2	360.8 ± 21.0*	397.3 ± 11.8	448.5 ± 11.2*
HR (beats/min)	388 ± 5	358 ± 9	401 ± 5	347 ± 15†	347 ± 12#	339 ± 8

AWTd, diastolic anterior wall thickness; LVDd, diastolic LV diameter; PWTd, diastolic posterior wall thickness; AWTs, systolic anterior wall thickness; LVDs, systolic LV diameter; PWTs, systolic posterior wall thickness; RWT, relative wall thickness; FS, fractional shortening; SV, stroke volume; HR, Heart rate. Values are means ± SEM; *p<0.05 vs. corresponding untreated; #p<0.05 vs. control WKY. †p<0.05 vs. control within treatment.

Table S2. Echocardiographic parameters of pulmonary artery and mitral valve blood flow

	**Controls**		**Pyridostigmine**		**Trandolapril**	
	**WKY**	**SHR**	**WKY**	**SHR**	**WKY**	**SHR**
**Pulmonary artery flow**						
Vmax ($m.s^{-1}$)	0.95 ± 0.03	1.01 ± 0.03	0.99 ± 0.02	1.04 ± 0.03	0.91 ± 0.03	1.09 ± 0.03 $^{\#\dagger}$
Vmean ($m.s^{-1}$)	0.38 ±0.01	0.36 ± 0.01	0.41 ± 0.01	0.37 ± 0.02	0.36 ± 0.01	0.36 ± 0.01
ATp (ms)	26.8 ± 0.8	24.6 ± 0.6	28.3 ± 1.4	25.8 ± 1.3	30.8 ± 1.1	27.0 ± 1.1
ETp (ms)	84.1 ± 1.4	87.2 ± 1.9	84.3 ± 1.4	89.5 ± 2.5	95.3 ± 2.8 *	88.7 ± 2.3
**Mitral valve**						
E ($m.s^{-1}$)	1.03 ± 0.03	1.18 ± 0.06	1.04 ± 0.04	1.14 ± 0.04	0.99 ± 0.03	1.06 ± 0.02
FTm (ms)	62.0 ± 1.5	63.6 ± 4.3	60.5 ± 1.3	65.9 ± 4.5	79.0 ± 4.0*	82.2 ± 3.5*$^{\#}$
IVCTm (ms)	12.5 ± 0.8	16.0 ± 1.7	12.8 ± 1.2	13.8 ± 2.0	11.1 ± 0.7	13.8 ± 0.5
ETm (ms)	62.5 ± 1.4	64.9 ± 1.4	59.7 ± 1.2	68.8 ± 1.4$^{\#\dagger}$	67.9 ± 1.9	65.8 ± 0.9
IVRTm (ms)	18.4 ± 0.9	26.6 ± 2.5$^{\#}$	17.8 ± 0.8	25.0 ± 1.6$^{\#\dagger}$	16.6 ± 0.4	16.3 ± 1.4*

Vmax, peak velocity; Vmean, mean velocity; ATp, acceleration time; ETp, ejection time in pulmonary artery; E, early ventricular filling velocity; FTm, filling time; IVCTm, isovolumic contraction time; ETm, ejection time; IVRTm, isovolumic relaxation time. Values are means ± SEM; *$p<0.05$ vs. corresponding untreated; $^{\#}p<0.05$ vs. control WKY. $^{\dagger}p<0.05$ vs. control within treatment.

Table S4. A list of differentially expressed proteins that were sorted into the proteome group P1

Protein ID	Gene	Protein name	SP/SC	ST/SC	WP/WC	WT/WC	SC/WC
Q712U5	Arpp19	cAMP-regulated phosphoprotein 19	-	SC	-	WT	SC
P06686	Atp1a2	Sodium/potassium-transporting ATPase subunit alpha-2	-	2.62	-	-	-2.02
P04218	Cd200	OX-2 membrane glycoprotein	SC	-	-	-	SC
P37397	Cnn3	Calponin-3	SC	SC	-	-	SC
BOBNA5	Cotl1	Coactosin-like protein	SC	SC	-	-	SC
O08557	Ddah1	N(G),N(G)-dimethylarginine dimethylaminohydrolase	-	-2.18	-	-	-3.20
A0A8I6AFJ7	Dnajc13	DnaJ heat shock protein family (Hsp40) member C13	SP	ST	-	-	WC
B2RZ77	Dpt	Dermatopontin	SC	SC	-	WT	SC
P08050	Gja1	Gap junction alpha-1 protein	-4.82	-	-	-	-2.20
F1LZJ4	Hyi	Putative hydroxypyruvate isomerase	-	ST	-	-	WC
D3ZLT6	Klhl31	Kelch-like family member 31	-	-2.23	-	-	2.07
Q6P6T9	Kpna2	Importin subunit alpha	SC	SC	-	-	SC
D4AD29	Lamtor4	Ragulator complex protein LAMTOR4	SP	-	-	-	WC
Q6MG66	Lsm2	LSM2 homolog, U6 small nuclear RNA and mRNA degradation-associated	SC	SC	-	WT	SC
D3Z952	Mfap2	Microfibril-associated protein 2	SP	ST	WC	WC	WC

A0A0G2K9S8	Mfap5	Microfibril-associated protein 5	SP	ST	-	WC	WC
M0RCF7	Myot	Myotilin	SC	SC	-	-	SC
D3ZLM5	Nhlrc2	NHL repeat containing 2	SP	-	-	-	WC
D4A4K6	Nrap	Nebulin-related-anchoring protein	-	-4.01	-	-	2.80
Q6AY63	Nudt5	ADP-sugar pyrophosphatase	SP	-	WC	WC	WC
F7FLB	Pgm2	Phosphoglucomutase	SC	SC	WP	-	SC
P29315	Rnh1	Ribonuclease inhibitor	2.65	2.83	-	-	-2.77
A0A8I6ASF5	Rpl4	60S ribosomal protein L4	SC	SC	-	WT	SC
B0BMX3	S100a16	S100 calcium binding protein A16	SC	SC	-	-	SC
A0A8I6AQI7	Serpinf2	Serpin family F member 2	-	SC	-	-	-2.11
M0R701	Tmem14c	Transmembrane protein 14C	SC	SC	-	-	SC
E9PT79	Tsn	Translin	SC	SC	WP	WT	SC
A0A8I5ZUK2	Ubap2l	Ubiquitin associated protein 2-like	SP	ST	-	-	WC

WC, protein expression detected only in samples from WKY control rats; WP, protein expression detected only in samples from WKY rats treated with pyridostigmine; WT, protein expression detected only in samples from WKY rats treated with trandolapril; SC, protein expression detected only in samples from control SHR rats; SP, protein expression detected only in samples from SHR rats administered pyridostigmine; ST, protein expression detected only in samples from SHR rats administered trandolapril

Table S5. A list of differentially expressed proteins that were sorted into the proteome group P2

Protein ID	Gene	Protein name	SP/SC	ST/SC	WP/WC	WT/WC
P16970	Abcd3	ATP-binding cassette subfamily D member 3	SC	-	-	-
D3ZD23	Abce1	ATP-binding cassette subfamily E member 1	-	SC	-	-
Q6MG08	Abcf1	ATP-binding cassette subfamily F member 1	2.25	-	-	-
M0RB66	Ace3	Angiotensin-converting enzyme	SP	-	-	-
Q9QZ81	Ago2	Protein argonaute-2	-	SC	2.04	-
P47197	Akt2	Rac-beta serine/threonine-protein kinase	-	SC	-	-
P61212	Arl1	ADP-ribosylation factor-like protein 1	-	SC	-	-
O88656	Arpc1b	Actin-related protein 2/3 complex subunit 1B	-	SC	-	-
A0A096MKE0	Asph	Aspartate-beta-hydroxylase	2.26	-	-	-
Q9ER24	Atxn10	Ataxin-10	-	SC	-	-
P07151	B2m	Beta-2-microglobulin	-	SC	-	-
B0BN74	Bag2	BAG cochaperone 2	SC	SC	-	-
D4A1J4	Bdh2	3-hydroxybutyrate dehydrogenase type 2	-	SC	-	-
Q8VHI8	Bnip1	Vesicle transport protein SEC20	SC	SC	-	-
D4A3I4	Btf3l4	Transcription factor BTF3	-	SC	-	-
P97829	Cd47	Leukocyte surface antigen CD47	SC	-	-	-
P70500	Cdipt	CDP-diacylglycerol—inositol 3-phosphatidyltransferase	-	SC	-	-
Q792H5	Celf2	CUGBP Elav-like family member 2	SC	SC	-	-
A0A8I6AEZ5	Clasp1	Cytoplasmic linker associated protein 1	SC	SC	-	-
P05371	Clu	Clusterin	-	SC	-	-

A0A8I5YBR2	Cops6	COP9 signalosome complex subunit 6	-	SC	-	WC
Q5U2U2	Crkl	Crk-like protein	-	SC	-	-
P67874	Csnk2b	Casein kinase II subunit beta	SC	SC	-	-
F7F0L0	Ctps1	CTP synthase	SP	-	-	-
A0A8I6G8I6	Cul2	Cullin 2	SC	SC	-	-
F7FNG5	Cul4a	Cullin 4A	SC	SC	-	-
D4A8H8	Cyfip1	Cytoplasmic FMR1-interacting protein	SC	SC	-	-
P61805	Dad1	Dolichyl-diphosphooligosaccharide—protein glycosyltransferase subunit DAD1	SP	-	-	-
A0A8I6GAE6	Ddx19b	RNA helicase	-	SC	-	-
A0A8I6GLR7	Dnah3	Dynein, axonemal, heavy chain 3	SC	SC	-	-
F1MAM6	Dnah8	Dynein, axonemal, heavy chain 8	SP	-	-	WT
P60905	Dnajc5	DnaJ homolog subfamily C member 5	SP	-	-	-
Q62871	Dync1i2	Cytoplasmic dynein 1 intermediate chain 2	-	SC	-	-
A0A8I5ZY32	Ehbp1l1	EH domain binding protein 1-like 1	SP	-	-	-
Q63184	Eif2ak2	Interferon-induced, double-stranded RNA-activated protein kinase	SC	-	-	-
Q6AYK8	Eif3d	Eukaryotic translation initiation factor 3 subunit D	SC	-	-	-
Q3KRD8	Eif6	Eukaryotic translation initiation factor 6	SC	-	-	-
B5DF91	Elavl1	ELAV-like protein 1	-	SC	-	-
A0A8I5ZYA8	Emc1	ER membrane protein complex subunit 1	-	SC	-	-

D3ZQL1	Emc7	ER membrane protein complex subunit 7	-	SC	-	WC
A0A8I6AHY5	Eppk1	Epiplakin 1	SC	SC	-	-
Q5U2Q7	Etf1	Eukaryotic peptide chain release factor subunit 1	-	SC	-	-
P16296	F9	Coagulation factor IX	SC	-	-	-
Q923V4	Fbxo6	F-box only protein 6	-	ST	-	-
Q9WUH4	Fhl1	Four and a half LIM domains protein 1	-	-2.29	-	-
P36365	Fmo1	Dimethylaniline monooxygenase [N-oxide-forming] 1	SC	SC	-	-
D3ZYS7	G3bp1	G3BP stress granule assembly factor 1	-	SC	-	-
F7F5P9	G3bp2	G3BP stress granule assembly factor 2	SC	SC	-	-
P97834	Gps1	COP9 signalosome complex subunit 1	-	SC	-	-
A0A8I6ASE8	H2afz	Histone H2A	SC	-	-	-
Q6AXM7	Hbs1l	HBS1-like protein	SC	SC	-	-
Q6P747	Hp1bp3	Heterochromatin protein 1-binding protein 3	-	SC	-	-
P97541	Hspb6	Heat shock protein beta-6	2.17	-	-	-
G3V824	Igf2r	Insulin-like growth factor 2 receptor	SC	SC	-	-
D3ZZX1	Inpp5a	Inositol polyphosphate-5-phosphatase A	-	SC	-	-
D3ZW59	Itgb1bp2	Integrin beta 1 binding protein 2	-	2.42	-	-
Q99PF5	Khsrp	Far upstream element-binding protein 2	-	SC	-	-
P37285	Klc1	Kinesin light chain 1	-	SC	-	-
B5DEP7	Snrpg	Small nuclear ribonucleoprotein G	SC	SC	-	-

A0A8I6A547	M6pr	Cation-dependent mannose-6-phosphate receptor-like	SC	SC	-	-
P15146	Map2	Microtubule-associated protein 2	SC	SC	-	-
F2Z3T4;F1M9N4	Mbnl1/2	Muscleblind-like protein 1/2	-	SC	-	-
P38062	Metap2	Methionine aminopeptidase 2	-	SC	-	-
P70490	Mfge8	Lactadherin	SC	-	-	-
P30904	Mif	Macrophage migration inhibitory factor	2.15	-	-	-
D4A1H7	Mul1	RING-type E3 ubiquitin transferase	SC	SC	-	-
Q9JLT0	Myh10	Myosin-10	-	SC	-	-
P12847	Myh3	Myosin-3	-	SC	-	-
F7F0L5	Myh7b	Myosin heavy chain 7B	SC	SC	-	-2.03
F1M7K3	Myl7	Myosin light chain 7	SC	SC	-	-
Q66H12	Naga	Alpha-N-acetylgalactosaminidase	-	SC	-	-
Q9Z2L9	Ndrg4	Protein NDRG4	SP	-	-	-
P42676	Nln	Neurolysin, mitochondrial	-	SC	-	-
Q9QUL6	Nsf	Vesicle-fusing ATPase	2.20	-	-	-
A0A8I5Y9V8	Nudcd3	NudC domain containing 3	SC	SC	-	-
P56558	Ogt	UDP-N-acetylglucosamine—peptide N-acetylglucosaminyltransferase 110 kDa subunit	-	SC	-	-
B2RYG6	Otub1	Ubiquitin thioesterase OTUB1	2.06	2.21	-	-
P0C5E3	Palld	Palladin	SC	SC	-	-
Q9HB97	Parva	Alpha-parvin	SC	-	-	-
Q99ML5	Pcyox1	Prenylcysteine oxidase 1	-	SC	-	-
G3V7W1	Pdcd6	Programmed cell death protein 6	-	SC	-	-
Q9Z1Z9	Pdlim7	PDZ and LIM domain protein 7	SC	SC	-	-

A0A8I6GJ67	Pin1	Peptidylprolyl cis/trans isomerase, NIMA-interacting1	SC	SC	-	-
P50411	Pp1r2	Protein phosphatase inhibitor 2	SP	-	-	-
D3ZLD7	Ppp2r3a	Protein phosphatase 2, regulatory subunit B, alpha	-	SC	-	-
Q64620	Ppp6c	Serine/threonine-protein phosphatase 6 catalytic subunit	SC	-	-	-
A0A8I6AAQ6	Prdx4	Peroxiredoxin-4	SC	SC	-	-
Q4V8C7	Prkra	Interferon-inducible double-stranded RNA-dependent protein kinase activator A	-	SC	-	-
Q4V8E2	Psmd14	Proteasome 26S subunit, non-ATPase, 14	-	SC	-	-
Q00438	Ptbp1	Polypyrimidine tract-binding protein 10	-	SC	-	-
P06302	Ptma	Prothymosin alpha	-2.16	SC	-	-
Q5PQJ6	Pycrl	Pyrroline-5-carboxylate reductase 3	SP	-	-	-
P35281	Rab10	Ras-related protein Rab-10	-	SC	-	-
A1L1J8	Rab5b	RAB5B, member RAS oncogene family	-	SC	-	-
Q27W01	Rbm8a	RNA-binding protein 8A	SC	SC	-	-
A0A8I5ZQQ9	Ak2	Adenylate kinase 2	SC	SC	-	-
Q32PX6	Rhog	Ras homolog family member G	SC	-	-	-
P62890	Rpl30	60S ribosomal protein L30	-	ST	-	-
A0A8I6ADZ2	Rpl37a	Uncharacterized protein	-2.34	-	-	-
P19944	Rplp1	60S acidic ribosomal protein P1	2.66	3.00	-	-
P62845	Rps15	40S ribosomal protein S15	-	-2.34	-	-
D3ZDU2	Rptor	Regulatory-associated protein of mTOR, complex 1	SC	-	WC	-
D3Z8L7	Rras	Ras-related protein R-Ras	SC	SC	WC	-
P50116	S100a9	Protein S100-A9	-	SC	-2.16	-3.19

A0A8I6GMG5	Sar1a	Secretion associated, Ras related GTPase 1A	-	SC	-	-
Q64380	Sardh	Sarcosine dehydrogenase, mitochondrial	-	SC	-	-
B2RZD1	Sec61b	Protein transport protein Sec61 subunit beta	-	SC	-	-
B3GNI6	Sept11	Septin-11	SC	-	-	-
B0BNF1	Sept8	Septin-8	-	SC	-	-
Q62773	Slc28a2	Sodium/nucleoside cotransporter 2	-	SC	-	-
P17136	Snrpb; Snrpn	Small nuclear ribonucleoprotein-associated protein B	SC	SC	-	-
B1H267	Snx5	Sortin nexin-5	SC	SC	-	-
Q99PV2	Stxbp3	Syntaxin binding protein 3	-	SC	-	-
A0A0G2JXP4	Tango2	Transport and Golgi organization 2 homolog	-	SC	WC	WC
E9PTK4	Thnsl1	Threonine synthase-like 1	SC	SC	-	-
P24155	Thop1	Thimet oligopeptidase	-	SC	-	-
A2VCX1	Tiprl	TIP41-like protein	-	SC	-	-
D3ZTX0	Tmed7	Transmembrane emp24 domain-containing protein 7	SC	SC	-	-
D3ZSG3	Tmod4	Tropomodulin 40	-	2.75	-	-
A0A8I6A1Z5	Tmx1	Thioredoxin-related transmembrane protein 1	-	SC	-	-
A0A8J8XJV6	Tnnc1	Troponin C1, slow skeletal and cardiac type	2.76	-	-	-
Q62760	Tomm20	Mitochondrial import receptor subunit TOM20 homolog	-	2.03	-	-
Q3KRD5	Tomm34	Mitochondrial import receptor subunit TOM34	SC	SC	-	-
Q75Q40	Tomm40	Mitochondrial import receptor subunit TOM40 homolog	-	SC	-	-
Q6PCT3	Tpd52l2	Tumor protein D54	-2.14	-	-	-

F1MA98	Tpr	Nucleoprotein TPR	SC	-	-	-
P02767	Ttr	Transthyretin	2.26	-	-	-
Q498E0	Txndc12	Thioredoxin domain-containing protein 12	SP	-	-	-
A0A8I6A0W6	Ube2o	Ubiquitin-conjugating enzyme E2O	SC	SC	-	-
Q2TL32	Ubr4	E3 ubiquitin-protein ligase UBR4	SC	SC	-	-
Q6BBI8	Ufc1	Ubiquitin-fold modifier-conjugating enzyme 1	SC	-	-	-
Q9JLA3	Uggt1	UDP-glucose:glycoprotein glucosyltransferase 1	-	SC	-	-
Q91Y78	Uchl3	Ubiquitin carboxyl-terminal hydrolase isozyme L3	-	ST	-	-
A0A0G2JZU8	Usp9y	Ubiquitinyl hydrolase 1	-	SC	-	-
A0A8I6GH80	Vps4b	Vesicle-fusing ATPase	-	SC	-	-
A0A8L2Q1U4	Wipi1	WD repeat domain, phosphoinositide-interacting 1	SC	SC	-	-
Q71LX6	Xirp2	Xin actin-binding repeat-containing protein 2	-	-2.48	-2.81	-2.21
D4A7U1	Zyx	Zyxin	-	SC	-	-

WC, protein expression detected only in samples from WKY control rats; WP, protein expression detected only in samples from WKY rats treated with pyridostigmine; WT, protein expression detected only in samples from WKY rats treated with trandolapril; SC, protein expression detected only in samples from SHR control rats; SP, protein expression detected only in samples from SHR rats administered pyridostigmine; ST, protein expression detected only in samples from SHR rats administered trandolapril

Table S6. A list of differentially expressed proteins that were sorted into the proteome group P3

Protein ID	Gene	Protein name	WP/WC	WT/WC	SC/WC
P14046	A1i3	Alpha-1-inhibitor 3	-	-	-2.02
A0A0G2K828	AABR07065774.1	Ig-like domain-containing protein	-	-	WC
D3ZCF8	Abca8a	ATP-binding cassette, subfamily A, member 8A	-	-	-2.45
P68136	Acta1	Actin, alpha skeletal muscle	-	-	3.55
P60711	Actb	Actin, cytoplasmic 1	-	WC	WC
Q63028	Add1	Alpha-adducin	-	-	WC
Q4KLH5	Agfg1	Arf-GAP domain and FG repeat-containing protein 1	-	WC	WC
P24090	Ahsg	Alpha-2-HS-glycoprotein	-	-	-2.26
O09178	Ampd3	AMP deaminase 3	WC	WC	WC
Q8R560	Ankrd1	Ankyrin repeat domain-containing protein 1	-	-	2.62
P11505	Atp2b1	Plasma membrane calcium-transporting ATPase 1	WC	-	WC
A0A8I6ATU9	Atxn2	Ataxin 2	-	-	WC
Q4V8K5	Brox	BRO1 domain-containing protein BROX	-	-	WC
P47727	Cbr1	Carbonyl reductase [NADPH] 1	-	-	-2.85
Q07969	Cd36	Platelet glycoprotein 4	-	-	-4.64
Q64244	Cd38	ADP-ribosyl cyclase/cyclic ADP-ribose hydrolase 1	-	-	WC
P40241	Cd9	CD9 antigen	-	-	-2,71
P10959	Ces1c	Carboxylesterase 1C	-	-	-2,85
P32038	Cfd	Complement factor D	-	WC	WC

F7ESI5	Cfhr1	Complement factor H-related 1	-	-	WC
Q68FT1	Coq9	Ubiquinone biosynthesis protein COQ9	-	-	-2.64
Q63135	Cr1l	Complement component receptor 1-like protein	-	-	WC
P50463	Csrp3	Cysteine and glycine-rich protein 3	-	-	2.34
P97694	Cyth1	Cytohesin-1	WC	WC	WC
E9PT29	Ddx17	RNA helicase	-	WC	WC
P63036	Dnaja1	DnaJ homolog subfamily A member 1	WC	-	WC
P63170	Dynll1	Dynein light chain 1	-	-	WC
A0A8I6G5P0	Eif2s2	Eukaryotic translation initiation factor 2 subunit beta	-	-	SC
Q63190	Emd	Emerin	-	-	WC
P15429	Eno3	Beta-enolase	-	-	-2.25
A0A8I6AQ74	ENSRNOG00000064086	Ig-like domain-containing protein	-	-	SC
A0A8I5ZV61	ENSRNOG00000065931	Ig-like domain-containing protein	-	WC	WC
A0A8I6AH36	ENSRNOG00000066971	Ig-like domain-containing protein	-	-	WC
Q6AYD4	Esam	Endothelial cell-selective adhesion molecule	-	-	WC
Q6AXX6	Fam213a	Peroxiredoxin-like 2A	-	-	SC
Q6AYQ3	Fars2	Phenylalanine—tRNA ligase	-	-	WC
O35115	Fhl2	Four and a half LIM domains protein 2	-	-	2.02
F7EYK0	Fkbp5	Peptidylprolyl isomerase	-	WC	WC
Q66HG4	Galm	Galactose mutarotase	-	WT	SC

Q63663	Gbp2	Guanylate-binding protein 1	WP	WT	SC
P62882	Gnb5	Guanine nucleotide-binding protein subunit beta-5	-	WC	WC
Q642B0	Gpc4	Glypican 4	-	-	WC
F1LML7	Hip1r	Huntingtin-interacting protein 1-related	WC	WC	WC
P0DMW1	Hspa1a	Heat shock 70 kDa protein 1B	-	-	-2.53
P85007	Ier3ip1	Immediate early response 3-interacting protein 1	-	WC	WC
A0A8I6GD94	Iggkvl-ps2	Ig-like domain-containing protein	-	-2.41	WC
Q5U3Z3	Isoc2	Isochorismatase domain-containing protein 2	-	-	-2.05
F2Z3T8	Gng5	Guanine nucleotide-binding protein subunit gamma	-	WC	WC
Q63910	LOC287167	Alpha globin	-	-	WC
Q66HR2	Mapre1	Microtubule-associated protein RP/EB family member 1	-	-	SC
Q9EPF2	Mcam	Cell surface glycoprotein MUC18	-	-	WC
D3ZE72	Metap1	Methionine aminopeptidase	-	-	WC
Q5HZE4	Mri1	Methylthioribose-1-phosphate isomerase	-	-	WC
P02564	Myh7	Myosin-7	-	-	2.20
D3ZSA9	Nomo1	Nodal modulator 1	-	WC	WC
O35264	Pafah1b2	Platelet-activating factor acetylhydrolase IB subunit alpha 2	WC	-	WC
Q9WUJ3	Pde4dip	Myomegalin	-	WC	WC

Q5XIP1	Pelo	Protein pelota homolog	-	WC	WC
Q5HZA6	Prepl	Prolyl endopeptidase-like	WC	-	WC
P13852	Prnp	Major prion protein	WC	-	WC
Q63797	Psme1	Proteasome activator complex subunit 1	-	-	-2.01
F7FJT3	Rad23a	UV excision repair protein RAD23	-	-	WC
O88350	Rbbp9	Serine hydrolase RBBP9	WC	WC	WC
D3ZFR9	Rdh13	Retinol dehydrogenase 13	-	-	WC
D3ZUY0	Rdh14	Retinol dehydrogenase 14	-	-	2.14
A0A8I6G8Z3	RGD1564696	Ig-like domain-containing protein	-	-	SC
Q0D2L6	Rragc; Rragd	Ras-related GTP-binding protein	WC	WC	WC
P05943	S100a10	Protein S100-A10	-	-	-2.52
D3ZM09	Sars2	Serine—tRNA ligase	-	-	-3.17
Q6AYR8	Scrn2	Secernin-2	-	-	SC
D4A071	Serhl2	Serine hydrolase-like 2	-	-	WC
P05545	Serpina3k	Serine protease inhibitor A3K	-	-	SC
P05544	Serpina3l	Serine protease inhibitor A3L	-	-	-2.31
Q63556	Serpina3m	Serine protease inhibitor A3M	-	-	WC
Q64268	Serpind1	Heparin cofactor 2	-	-	WC
D4A081	Setdb1	SET domain bifurcated histone lysine methyltransferase 1	-	WC	WC
Q5XIF9	Slc25a34	Solute carrier family 25 member 34	-	WC	WC
D4A678	Spta1	Spectrin, alpha, erythrocytic 1	-	-	4.13
A0A8I6AWM3	Sptb	Spectrin beta chain	-	-	-3.08

P15589	Sts	Steryl-sulfatase	-	WC	2.27
A0A8I6ADE8	Sync	Syncoilin, intermediate filament protein	WC	WC	WC
A0A8I5ZJB9	Syne1	Spectrin repeat containing, nuclear envelope 1	-	2.16	WC
G3V9G5	Synm	RCG24674, isoform CRA_b	-	-	-3.60
A0A0G2K598	Tacc2	Transforming, acidic coiled-coil-containing protein 2	-	WC	WC
D3ZJO0	Tmem205	Transmembrane protein 205	WC	-	WC
Q5FVN2	Tmem41b	Transmembrane protein 41B	WC	WC	WC
Q5XIP9	Tmem43	Transmembrane protein 43	-	-	WC
M0R402	Tmx3	RCG20581, isoform CRA_a	-	-	WC
F7EYC4	Tom1	Target of myb1 membrane trafficking protein	-	-	WC
F7FE56	Tpd52l1	TPD52 like 1	-	-	3.43
P68370	Tuba1a	Tubulin alpha-1A chain	-	-	WC
B5DEH4	Uap1l1	UDP-N-acetylglucosamine pyrphosphorylase 1-like 1	-	-	WC
P62255	Ube2g1	Ubiquitin-conjugating enzyme E2 G1	WC	WC	WC
F7FPV0	Umps	Uridine 5-monophosphate synthase	WC	-	WC
Q9R085	Usp15	Ubiquitin carboxyl-terminal hydrolase 15	WC	WC	-
F1LSM0	Usp24	Ubiquitin-specific peptidase 24	-	-	SC
B2GUZ1	Usp4	Ubiquitin carboxyl-terminal hydrolase 4	-	WC	-
G3V7L1	Utrn	Utrophin	-	-	WC

A0A8I6A4A2	Vps13a	Vacuolar protein sortin 13 homolog A	-	-	WC
F1M8H2	Wars2	Tryptophan—tRNA ligase	-	-	WC
Q4KM49	Yars	Tyrosine—tRNA ligase, cytoplasmic	WC	-	-
D4AE17	Zak	Mitogen-activated protein kinase kinase kinase 20	-	-	WC
P20759	Ig gamma-1, Ighg1	Ig gamma-1chain C region	-	-	WC
P01836	Ig kappa, Igkc	Ig kappa chain C region, A allele	-	-	SC

WC, protein expression detected only in samples from WKY control rats; WP, protein expression detected only in samples from WKY rats treated with pyridostigmine; WT, protein expression detected only in samples from WKY rats treated with trandolapril; SC, protein expression detected only in samples from SHR control rats
