# Supplementary material for: Modulation of left ventricular hypertrophy in spontaneously hypertensive rats by acetylcholinesterase and ACE inhibitors: physiological, biochemical, and proteomic studies
Source: Front Cardiovasc Med. 2024 Sep 16;11:1390547. doi: 10.3389/fcvm.2024.1390547 (PMC11443425; doi:10.3389/fcvm.2024.1390547)
Supplement: Supplementary file 4 [file Datasheet3.pdf]

Supplementary Material

Supplementary Results

1.1 General strategy for detailed assessment of proteome changes

In all seven pairwise comparisons, a total of 666 alterations were found in 285 proteins (Suppl. Table S3). The 131 alterations were found in the pairwise comparison of SHR and WKY control rats (SC/WC), the pairwise comparison with the largest number of alterations (Suppl. Table S3). Both PYR and TRA elicited significantly more alterations in SHR rats compared to WKY rats (Suppl. Table S3). Moreover, the proteomic profiles of SHR rats and WKY rats treated with PYR or TRA differed markedly (Suppl. Table S3). Treatment with PYR or TRA altered only a few proteins in both strains in the same trend (Cops6, Emc7, Myh7b, Rptor, Rras, S100a9, Tango2, Xirp2) or in the opposite trend (Dpt, Lsm2, Mfap2, Mfap5, Nudt5, Pgm2, Rpl4-ps1, Tomm34, Tsn) (Suppl. Table S3). In contrast, many changes were found in SP/WP and ST/WT comparisons indicating the different effects of PYR or TRA on the proteome of these two strains. In SHR rats, PYR or TRA altered the expression of 93 and 128 proteins, respectively (Suppl. Table S3), suggesting that TRA is a more efficient agent for regulating protein levels. The expression levels of 55 proteins were simultaneously altered by both inhibitors with the same trend (Suppl. Table S3). This indicates that the effects of these inhibitors on protein expression partially overlap.

To clearly determine the effect of PYR or TRA on the changes between the proteomes of WKY rats and SHR rats, we categorized the altered proteins into three proteome groups: 1) proteome group P1 includes proteins whose levels were differentially altered in the pairwise SC/WC comparison and in at least one of the pairwise SP/SC or ST/SC comparisons; 2) proteome group P2 includes proteins whose levels were differentially altered in the pairwise SP/SC and/or ST/SC comparisons without differential alteration in the pairwise SC/WC comparison; 3) proteome group P3 comprises proteins whose levels were differentially altered in the pairwise SC/WC comparison, but no differential changes were present in the pairwise SP/SC and/or ST/SC comparison. Proteome group P1 represents proteome differences between WKY and SHR control rats that are increasingly promoted or conversely suppressed by PYR or TRA. Proteome group P2 represents proteome differences that are induced by PYR or TRA and only occur in SHR rats. Proteome group P3 represents proteome differences between control WKY and SHR rats that were not affected by PYR and TRA. These three proteome groups were further analyzed with respect to biological processes mediated by differentially altered proteins. The STRING database to analyze functional protein association networks (Suppl. Figures S3-S5). Each proteome group was analyzed using the GO (Gene Ontology) tool DAVID (The Database for Annotation, Visualization and Integrated Discovery) to determine the biological processes (Suppl. Fig. S6). The protein data sets were sorted according to their relationships between the biological processes. The altered proteins were related to protein turnover, RNA processing, cytoskeletal dynamics, muscle function and regulation of signaling pathways, including insulin, mTOR and TGFβ signaling.

1.2. Gene ontology analysis of proteins in proteomic groups P1-P3

The first proteome group P1 comprises 30 proteins with differential expression between control WKY and SHR rats, and these differences were progressively promoted or conversely suppressed by PYR or TRA. GO analysis of the proteins in the first proteome group P1 using the DAVID tool revealed the effect of PYR or TRA on assembly of blood vessel morphogenesis and the regulation of TGF-β production with p-values 1.00×10^{-1} and 2.80×10^{-2}, respectively (Suppl. Figure S6A). The other three

biological processes, including phagocytosis, cytoskeletal dynamics, and nuclear transport, were found by analyzing the protein interactions in the STRING database (Suppl. Figure S3).

The second proteome group P2 includes 136 proteins with similar expression in WKY and SHR control rats, but different expression in SHR control rats and those administered PYR or TRA. Only eleven proteins from the whole proteome group were detected with alterations induced by PYR or TRA in WKY rats (Suppl. Table S3 and S5). This means that PYR and TRA act as regulators of gene expression and protein turnover with different effects on these two rat phenotypes, although there were no differences in the levels of these proteins under control conditions in WKY and SHR rats. GO analysis performed with the DAVID tool showed the effect of PYR or TRA on several biological processes related to translation (p-values ranging from 5.40 x 10^{-4} to 1.00 x 10^{-1}), protein transport (p-values ranging from 4.40 x 10^{-3} to 9.10 x 10^{-2}), protein turnover (p-values ranging from 2.30 x 10^{-2} to 9.10 x 10^{-2}), RNA splicing (p-values ranging from 3.80 x 10^{-3} to 9.70 x 10^{-2}), regulation of gene expression by small RNA (p-values ranging from 9.10 x 10^{-2} to 1.00 x 10^{-1}), organization of the cytoskeleton (p-values ranging from 7.80 x 10^{-3} to 8.30 x 10^{-2}), cell organization (p-values ranging from 2.30 x 10^{-2} to 9.00 x 10^{-2}), muscle contraction (p-values ranging from 4.70 x 10^{-2} to 1.00 x 10^{-1}), immune response (p-value 8.50 x 10^{-2}) or response to insulin (p-values ranging from 4.10 x 10^{-2} to 5.80 x 10^{-2}) (Suppl. Figure S6B).

The third proteome group P3 comprises 101 proteins with differential expression between control WKY and SHR control rats and no effects of PYR and TRA. GO analysis performed with the DAVID tool revealed that PYR and TRA had no effect on the expression of some proteins involved in phagocytosis (p-values between 5.00 x 10^{-3} and 8.10 x 10^{-2}), immune response (p-values between 9.60 x 10^{-3} and 4.00 x 10^{-2}), the response to stimuli (p-values between 3.40 x 10^{-2} and 7.10 x 10^{-2}), organization of the cytoskeleton (p-values between 5.80 x 10^{-2} and 7.70 x 10^{-2}), enzymatic activity (p-values between 1.90 x 10^{-3} and 5.30 x 10^{-2}) and translation (p-values between 1.30 x 10^{-2} and 5.20 x 10^{-2}) (Suppl. Figure S6C).

1.3 Protein-protein associations in proteome group P1

The protein-protein associations identified by the STRING database are shown in Supplementary Figure S3. One protein pair, Lamtor4 and Nhlrc2, exhibited the same expression pattern when both proteins were detected only in WKY control rats compared to SHR control rats and their levels were restored in SHR rats by PYR (Suppl. Table S4 and Suppl. Figure S7). Lamtor4 is a component of the Rag-Regulator complex and an essential regulator of lysosomes, which plays an indispensable role in the degradative processes of phagocytosis (1). Nhlrc2 has been found to be involved in the initiation of localized RhoA inhibition, enabling Rac1-driven cytoskeletal rearrangement during the initiation of phagocytosis, including the control of actin polymerization and filopodia formation (2). In our study, several proteins (Myot, Nrap, Klhl31, Cotl1 and Cnn3) associated with actin cytoskeleton organization were found to be differentially altered. Myotilin (Myot), coactosin-like protein 1 (Cotl1) and calponin 3 (Cnn3) were only detected in SHR control rats compared to WKY control rats and were simultaneously undetetable in SHR rats receiving PYR or TRA (Suppl. Table S4 and Fig. S7). The levels of nebulin-related anchoring protein (Nrap) and Kelch-like protein 31 (Klhl31) were quantitatively upregulated in SHR control rats and down-regulated by TRA in SHR rats (Suppl. Table S4 and Suppl. Figure S7). Calponin 3 induces actin polymerization and its knockdown leads to a change in contractility (3, 4). Cotl1 is an actin-binding protein and regulates lamellipodial dynamics by protecting F-actin from cofilin-mediated depolymerization (5). Myotilin binds to F-actin, filamin C, α-actinin and other components at the sarcomeric Z-disk, the boundaries that crosslink thin actin filaments in an antiparallel fashion, and controls the assembly of sarcomeres, the basic contractile units of striated muscle cells (6). Nrap, which is expressed in the heart,

is an actin- and filamin C-binding cytoskeletal protein (7). Klhl31 preserves muscle integrity by maintaining functional filamin C protein levels. The large filamin C aggregates that reduce Z-disk stability were observed in Klhl31 knockout mice (8). In our study, filamin C (UniProt ID D3ZHA0), α-actinin-1 (UniProt ID Q9Z1P2), α-actinin-2 (UniProt ID A0A8I5ZN92) and α-actinin-4 (UniProt ID Q9QXQ0) were detected and no alterations in their levels were found in all seven pairwise comparisons (Suppl. Table S3). However, our data suggest that SHR and WKY rats may differ markedly in myocardial contractility, which is associated with changes in actin polymerization, filamin C aggregate formation and Z-disk stability, and that these differences may be regulated by PYR and TRA.

Three altered proteins, microfibril-associated protein 2 (MFAP2, MAGP1), microfibril-associated protein 5 (MFAP5, MAGP2) and dermatopontin (Dpt) are associated with the dynamic structural network of the extracellular matrix (9, 10). Mfap2 and Mfap5 were only detected in WKY control rats compared to SHR control rats and both treatments caused restoration of their expression in SHR rats (Suppl. Table S4 and Figure S7). In contrast, dermatopontin was only detected in SHR control rats compared to WKY control rats and was simultaneously undetectable in SHR rats receiving PYR or TRA (Suppl. Table S3 and Fig. S7). Both MFAPs are associated with fibrillin in vertebrate extracellular matrix microfibrils, which act as structural support of tissues and control the availability of growth factors, mainly members of the TGFβ superfamily (9). TGFβs are co-secreted with latent TGFβ-binding protein (LTBP) in an inactive latent form that binds to fibrillin and requires activation to release active TGFβ (9). It has been suggested that MFAP2 binds to the same region of fibrillin as the latent TGFβ-LTBP complex, blocking its binding, which impairs TGFβ activity and signaling (9, 11). MFAPs can also directly bind the active form of TGFβ and could serve to attenuate or terminate TGFβ signaling by removing excess TGFβ (9), but TGFβ1 has been confirmed to retain signaling activity when complexed with MFAP2 (11). Inactivation of the MFAP2 gene and its subsequent absence led to an increase in the total amount TGFβ stored in the extracellular matrix, as TGFβ could no longer bind to fibrillin via MFAP2, resulting in dysregulation of the TGFβ signaling system (11). Dermatopontin has been shown to regulate the interaction between decorin and TGFβ and increase the biological activity of TGFβ (10). In our study, we could not detect TGFβ at all, but the biological processes found in the GO analysis using the DAVID tool indicated positive regulation of TGFβ production and blood vessel morphogenesis (Suppl. Fig. S7). Both biological processes are linked, as TGFβ signaling is involved in the spatial and temporal regulation of cardiac and vascular morphogenesis, and dysregulated TGFβ signaling causes vascular pathologies and cardiovascular diseases such as arteriovenous malformations or aneurysms (12). Cd200 has been shown to modulate TGFβ production (12, 14). In our study, Cd200 was only detected in SHR control rats compared to WKY control rats and was suppressed by administration of PYR to SHR rats (Suppl. Table S3 and Fig. S7). Serpinf2, also called α-2-antiplasmin, is involved in TGFβ1 production, as the amount of mRNA for TGFβ1 was decreased in primary cultured fibroblasts obtained from the normal skin of $\alpha 2AP^{-/-}$ C57BL/6J mice compared with fibroblasts from $\alpha 2AP^{+/+}$ mice (15). In our study, Serpinf2 levels were slightly decreased in SHR control rats compared to WKY control rats and administration of TRA to SHR rats resulted in undetectable Serpinf2 levels (Suppl. Table S4 and Figure S7). While Cd200 and Sefpinf2 are proteins upstream TGF signaling, the protein Gja1 (alternatively Cx43, Cxn43) is downstream of TGF signaling, as TGFβ1 increases the expression of Gja1 and modulates gap junction formation (16, 17). In our study, Gja1 levels were slightly decreased in SHR control rats compared to WKY control rats and significantly decreased in SHR rats administered PYR compared to SHR control rats (Suppl. Table S4 and Suppl. Figure S7). These data suggest that PYR may reduce Cd200 expression and subsequent TGFβ production, resulting in decreased Gja1 expression. It has been shown that the loss of Gja1 leads to embryonic vascular dysmorphogenesis, but alternative connexin isoforms (Cx45, Cx40) can compensate for this. However, they apparently only partially fulfill the function of Gja1/Cx43 in the heart (18, 19). In our study, other connexin isoforms (Cx45, Cx40, Cx32) were not

detected at all. Their levels could be investigated to clarify whether PYR induces changes in the expression of other connexins and the results could be related to TGF production and functional properties influenced by connexins (e.g., electrical impulse propagation or conduction of electrical impulses).

1.4 Protein-protein associations in proteome groups P2 and P3

The interactions and associations between the proteins sorted into proteome group P2 and P3 (Suppl. Table S5 and S6) were determined using the STRING database and are shown in Supplementary Figures S4 and S5, respectively. Many protein alterations from proteome group P2 are mentioned in the Results section of the main text.

The RNA-binding proteins, Elavl1, Rbma8a and Celf2, which are required for RNA stability and splicing (20-22), were undetectable in SHR rats administered PYR or TRA (Figure 8 and Suppl. Table S5), suggesting that both inhibitors significantly affect alternative RNA splicing and generate different alternative splicing profiles that influence biological processes.

Several differentially expressed proteins were found to be involved in the formation of thin filaments in the sarcomere, a contractile unit in the muscle fibers. The sarcomeric myosins Myh3, Myh7b and Myl7 as well as troponin C (Tnnc1) (23) were differentially expressed in SHR rats administered PYR or TRA compared to SHR control rats (Suppl. Figure S7 and Suppl. Table S5).

Increased insulin-like growth factor signaling is associated with the development of pathological cardiac hypertrophy and tumorigenesis (24) and suppression of IGF2R signaling may help prevent the transition from physiological hypertrophy to pathological hypertrophy (25). Increased expression of cardiac IGF2 and IGF2R has been demonstrated during the fetal and postnatal period in SHR rats (26). Our study showed that the level of insulin-like growth factor 2 receptor (IGF2R) was undetectable in SHR rats treated with PYR or TRA in contrast to SHR control rats (Suppl. Figure S7 and Suppl. Table S5). This suggests that both inhibitors may suppress the development of pathophysiological cardiac hypertrophy.

References

1. Shen, K, Sidik, H, and Talbot, WS. The Rag-Ragulator Complex Regulates Lysosome Function and Phagocytic Flux in Microglia. *Cell Rep* (2016) 14(3):547-59. doi:10.1016/j.celrep.2015.12.055

2. Haney, MS, Bohlen, CJ, Morgens, DW, Ousey, JA, Barkal, AA, Tsui, CK, et al. Identification of phagocytosis regulators using magnetic genome-wide CRISPR screens. *Nat Genet* (2018) 50(12):1716-27. doi:10.1038/s41588-018-0254-1

3. She, Y, Li, C, Jiang, T, Lei, S, Zhou, S, Shi, H, et al. Knockdown of CNN3 Impairs Myoblast Proliferation, Differentiation, and Protein Synthesis via the mTOR Pathway. *Front Physiol* (2021) 12(659272. doi:10.3389/fphys.2021.659272

4. Ciuba, K, Hawkes, W, Tojkander, S, Kogan, K, Engel, U, Iskratsch, T, et al. Calponin-3 is critical for coordinated contractility of actin stress fibers. *Sci Rep* (2018) 8(1):17670. doi:10.1038/s41598-018-35948-6

5. Li, G, Yin, Y, Chen, J, Fan, Y, Ma, J, Huang, Y, et al. Coactosin-like protein 1 inhibits neuronal migration during mouse corticogenesis. *J Vet Sci* (2018) 19(1):21-26. doi:10.4142/jvs.2018.19.1.21

6. Kostan, J, Pavsic, M, Puz, V, Schwarz, TC, Drepper, F, Molt, S, et al. Molecular basis of F-actin regulation and sarcomere assembly via myotilin. *PLoS Biol* (2021) 19(4):e3001148. doi:10.1371/journal.pbio.3001148

7. Truszkowska, GT, Bilinska, ZT, Muchowicz, A, Pollak, A, Biernacka, A, Kozar-Kaminska, K, et al. Homozygous truncating mutation in NRAP gene identified by whole exome sequencing in a patient with dilated cardiomyopathy. *Sci Rep* (2017) 7(1):3362. doi:10.1038/s41598-017-03189-8

8. Prill, K, and Dawson, JF. Assembly and Maintenance of Sarcomere Thin Filaments and Associated Diseases. *Int J Mol Sci* (2020) 21(2):doi:10.3390/ijms21020542

9. Craft, CS, Broekelmann, TJ, and Mecham, RP. Microfibril-associated glycoproteins MAGP-1 and MAGP-2 in disease. *Matrix Biol* (2018) 71-72(100-11. doi:10.1016/j.matbio.2018.03.006

10. Huang, H, Hao, Z, Long, L, Yin, Z, Wu, C, Zhou, X, et al. Dermatopontin as a potential pathogenic factor in endometrial cancer. *Oncol Lett* (2021) 21(5):408. doi:10.3892/ol.2021.12669

11. Broekelmann, TJ, Bodmer, NK, and Mecham, RP. Identification of the growth factor-binding sequence in the extracellular matrix protein MAGP-1. *J Biol Chem* (2020) 295(9):2687-97. doi:10.1074/jbc.RA119.010540

12. Goumans, MJ, and Ten Dijke, P. TGF-beta Signaling in Control of Cardiovascular Function. *Cold Spring Harb Perspect Biol* (2018) 10(2):doi:10.1101/cshperspect.a022210

13. Holmannova, D, Kolackova, M, Kondelkova, K, Kunes, P, Krejsek, J, and Ctirad, A. CD200/CD200R paired potent inhibitory molecules regulating immune and inflammatory responses; Part II: CD200/CD200R potential clinical applications. *Acta Medica (Hradec Kralove)* (2012) 55(2):59-65. doi:10.14712/18059694.2015.56

14. Kotwica-Mojzych, K, Jodlowska-Jedrych, B, and Mojzych, M. CD200:CD200R Interactions and Their Importance in Immunoregulation. *Int J Mol Sci* (2021) 22(4):doi:10.3390/ijms22041602

15. Kanno, Y, Kuroki, A, Okada, K, Tomogane, K, Ueshima, S, Matsuo, O, et al. Alpha2-antiplasmin is involved in the production of transforming growth factor beta1 and fibrosis. *J Thromb Haemost* (2007) 5(11):2266-73. doi:10.1111/j.1538-7836.2007.02745.x

16. Liu, W, Zhang, D, Li, X, Zheng, L, Cui, C, Cui, Y, et al. TGF-beta1 facilitates cell-cell communication in osteocytes via connexin43- and pannexin1-dependent gap junctions. *Cell Death Discov* (2019) 5(141. doi:10.1038/s41420-019-0221-3

17. Zeitz, MJ, Calhoun, PJ, James, CC, Taetzsch, T, George, KK, Robel, S, et al. Dynamic UTR Usage Regulates Alternative Translation to Modulate Gap Junction Formation during Stress and Aging. *Cell Rep* (2019) 27(9):2737-47 e5. doi:10.1016/j.celrep.2019.04.114

18. Payne, LB, Tewari, BP, Dunkenberger, L, Bond, S, Savelli, A, Darden, J, et al. Pericyte Progenitor Coupling to the Emerging Endothelium During Vasculogenesis via Connexin 43. *Arterioscler Thromb Vasc Biol* (2022) 42(4):e96-e114. doi:10.1161/ATVBAHA.121.317324

19. Gros, D, Dupays, L, Alcolea, S, Meysen, S, Miquerol, L, and Theveniau-Ruissy, M. Genetically modified mice: tools to decode the functions of connexins in the heart-new models for cardiovascular research. *Cardiovasc Res* (2004) 62(2):299-308. doi:10.1016/j.cardiores.2004.02.010

20. David, G, Reboutier, D, Deschamps, S, Mereau, A, Taylor, W, Padilla-Parra, S, et al. The RNA-binding proteins CELF1 and ELAVL1 cooperatively control the alternative splicing of CD44. *Biochem Biophys Res Commun* (2022) 626(79-84. doi:10.1016/j.bbrc.2022.07.073

21. Liu, Q, Fang, L, and Wu, C. Alternative Splicing and Isoforms: From Mechanisms to Diseases. *Genes (Basel)* (2022) 13(3):doi:10.3390/genes13030401

22. Beauchamp, MC, Alam, SS, Kumar, S, and Jerome-Majewska, LA. Spliceosomopathies and neurocristopathies: Two sides of the same coin? *Dev Dyn* (2020) 249(8):924-45. doi:10.1002/dvdy.183

23. England, J, and Loughna, S. Heavy and light roles: myosin in the morphogenesis of the heart. *Cell Mol Life Sci* (2013) 70(7):1221-39. doi:10.1007/s00018-012-1131-1

24. Feng, CC, Pandey, S, Lin, CY, Shen, CY, Chang, RL, Chang, TT, et al. Cardiac apoptosis induced under high glucose condition involves activation of IGF2R signaling in H9c2 cardiomyoblasts and streptozotocin-induced diabetic rat hearts. *Biomed Pharmacother* (2018) 97(880-85. doi:10.1016/j.biopha.2017.11.020

25. Chu, CH, Tzang, BS, Chen, LM, Kuo, CH, Cheng, YC, Chen, LY, et al. IGF-II/mannose-6-phosphate receptor signaling induced cell hypertrophy and atrial natriuretic peptide/BNP expression via Galphaq interaction and protein kinase C-alpha/CaMKII activation in H9c2 cardiomyoblast cells. *J Endocrinol* (2008) 197(2):381-90. doi:10.1677/JOE-07-0619

26. Engelmann, GL, Boehm, KD, Haskell, JF, Khairallah, PA, and Ilan, J. Insulin-like growth factors and neonatal cardiomyocyte development: ventricular gene expression and membrane receptor variations in normotensive and hypertensive rats. *Mol Cell Endocrinol* (1989) 63(1-2):1-14. doi:10.1016/0303-7207(89)90076-2
